# GC–MS, quantum mechanics calculation and the antifungal activity of river red gum essential oil when applied to four natural textiles

**DOI:** 10.1038/s41598-023-45480-x

**Published:** 2023-10-25

**Authors:** Ayman S. Taha, Ibrahim H. M. Ibrahim, Wael A. A. Abo-Elgat, Ahmed Abdel-Megeed, Mohamed Z. M. Salem, Mamoun S. M. Abd El-Kareem

**Affiliations:** 1 Conservation Department, Faculty of Archaeology, Aswan University, Aswan, 81528 Egypt; 2 Restoration Department, High Institute of Tourism, Hotel Management and Restoration, Abu Qir, Alexandria, Egypt; 3 Department of Plant Protection, Faculty of Agriculture (Saba Basha), Alexandria University, Alexandria, 21531 Egypt; 4 Forestry and Wood Technology Department, Faculty of Agriculture (EL-Shatby), Alexandria University, Alexandria, 21545 Egypt; 5 Atomic and Molecular Physics Unit, Experimental Nuclear Physics Department, Nuclear Research Centre, Egyptian Atomic Energy Authority, Inshas, Cairo, 13759 Egypt

**Keywords:** Microbiology, Chemistry, Materials science

## Abstract

The most important uses of old fabrics include clothing, mummification, and bookbinding. However, because they are predominantly constructed of natural materials, they are particularly susceptible to physical and chemical deterioration brought on by fungi. The treatments that are typically used to preserve old textiles focus on the use of synthetic fungicides, which have the potential to be dangerous for both human health and the environment. Essential oils (EOs), which are safe for the environment and have no negative effects on human health, have been widely advocated as an alternative to conventional antifungals. Four natural fabrics—linen, cotton, wool, and silk—were utilized in the current work. The extracted EO from leaves of river red gum (*Eucalyptus camaldulensis* Dehnh.) were prepared at 125, 250, and 500 µL/L. *Aspergillus flavus*, *Fusarium culmorum* and *Aspergillus niger* were inoculated separately into the treated four fabrics with the EO at concentrations of 125, 250, and 500 µL/L or the main compounds (spathulenol and eucalyptol) at the concentrations of 6, 12, 25, and 50 µL/L and were then compared to the un-treated samples. GC–MS was used to analyze the EO chemical composition, while visual observations and scanning electron microscopic (SEM) were used to study the fungal growth inhibition. Spathulenol (26.56%), eucalyptol (14.91%), and *p*-cymene (12.40%) were the principal chemical components found in *E. camaldulensis* EO by GC–MS. Spathulenol molecule displayed the highest electrostatic potential (ESP) compared with the other primary compound, as calculated by quantum mechanics. In the untreated textile samples, SEM analysis revealed substantial proliferation of hyphae from *A. flavus*, *F. culmorum*, and *A. niger*. The fungal growth was completely inhibited at a concentration of 500 µL/L from the EO. Both eucalyptol and spathulenol completely inhibited the formation of the fungal spores at a concentration of 50 µL/L, although eucalyptol was more effective than spathulenol across the board for all four textiles. The results support *E. camaldulensis* EO functionalized textiles as an effective active antifungal agent.

## Introduction

The world was aware of the widespread use of textiles for a variety of tasks, the most significant of which was the production of clothing, mummification, and the binding of antiquarian books^1–4^. The most significant of which was the binding of the Description of Egypt collection books kept in various Egyptian museums and libraries^5,6^.

There are numerous sources for the raw materials that can be used in the textile industry. Natural fibers come from plants (cotton, linen, and bast fibers)^7–10^, from animal or natural protein fibers (wool, silk)^9,11^, and natural mineral fibers (asbestos)^12^.

Microfungi have the potential to deteriorate textile materials^13,14^. The following fungi have been found to be destructive to textile materials in the soil and the air are: *Aspergillus*, *Penicillium*, *Alternaria*, *Cladosporium*, *Fusarium*, *Trichoderma*, etc.^15,16^. Microorganisms break down fiber and cloth on average, resulting in a loss of bulk and mechanical strength^17^. Fungi, which cause the enzymatic degradation of cellulose and penetrate the secondary wall, are primarily responsible for the breakdown of cellulose and cellulosic textile substrates^18–20^.

Nowadays, there is high demand for fabrics with functional or specialized finishes generally, but antimicrobial treatments, in particular, to protect people from microorganisms^21–24^. Therefore, to prevent toxic emissions from chemicals and enhance antimicrobial activity, various additives, such as natural pigments as dyes, were utilized in the textile fabrics (cotton, wool, silk, and nylon) for clean and environmentally friendly technologies^25^. Silver nanoparticles (AgNPs)-treated textiles have been found to be an environmentally friendly preservative that boosts product durability and inhibits fungal development^15^. The maximum activity against *Stemphylium solani* was seen in flax pulp treated with Paraloid B-72 + Ag NP (1%)^26^. With the addition of copper silicate, the created biofunctionalization of nonwovens with polypropylene and biodegradable polylactide had outstanding antimicrobial capabilities against *Escherichia coli* and *Staphylococcus aureus,* and *Candida albicans*^27^. They also produced biodegradable nonwoven textiles^27^. In comparison to untreated fibers, cotton textiles grafted with polyacrylic acid that was immobilized organic phosphorus monomer demonstrated notable flame-retardant capabilities and altered the mechanical behavior of the cotton^28^. The polyester and viscose nonwovens were combined with triclosan-loaded microparticles to create a finished product that was very effective against *S. aureus* and *K. pneumoniae*^29^.

Natural substances including essential oils (EOs), phenolic compounds, and flavonoid compounds have been intensively studied for their antimicrobial activities when applied to organic materials like wood, paper, textiles, and parchment^10,30–32^. Textiles have become more environmentally and human-friendly by using bioactive materials and dyes as finishing agents^33–36^.An efficient natural antibacterial and antifungal finish for cotton textiles has been created using the bioactive compounds of Neem plants combined with extracted *Aloe vera* gel^37^. Treated cotton fabrics especially in medical textiles with lavender, thyme and clove EOs had significant antibacterial activity against, *S. aureus* and *Escherichia coli*^38^. Numerous EOs and their individual components have been successfully used to combat a variety of pathogenic bacteria through their promising anti-quorum sensing and anti-biofilm forming properties^39–43^.

The growth of fungus mycelium and spore germination was inhibited by a number of naturally occurring substances used for textile dyeing^44^. *Mahonia napaulensis* dyed textile pieces showed effective antifungal properties against *Colletotrichum capsici*, *Leptosphaerulin trifoli*, *Alternaria brassicicola*, and *Helminthosporium solani* in both methanolic and aqueous extracts^45^. Neem and Mexican daisy extracts were used to create an antimicrobial finish on cotton fabric. Both direct application and microencapsulation were used, and the results showed that the microencapsulated herbal extracts had excellent resistance to microorganisms even after 15 washings^46^. The treated 100% cotton fabrics with *Aloe vera* methanol extract exhibited antimicrobial activity against the *S. aureus*^47^. Incorporated lime EO microcapsules into the cotton fabric demonstrated strong antibacterial action against *E. coli*, *Bacillus cereus*, *Salmonella typhimurium*, and *S. aureus*^48^.

In order to create novel and useful applications in fields related to human health, agriculture, and the environment without causing the same secondary effects that chemicals may cause, it is important to better understand the chemical complexity and biological properties of EOs^49–52^. The diverse biological features of the EOs are conferred by their substantial chemical composition and considerable variance^53–57^. The main components of the EOs, however, are not the only agents in charge of these properties^58–60^. Textile fabric treated with *Rosmarinus officinalis* and *Citrus sinensis* EOs had promising antifungal efficacy against *Aspergillus niger*, *A. flavus*, *C. albicans*, *Trichoderma viride* and *Epidermophyton floccosum*^61^. Tea, and eucalyptus EOs were used to treat cotton fabrics, and promising antimicrobial effectiveness was seen^47^. *Trichophyton interdigitale* was fully inhibited from growing on cotton fabrics by the application of EOs from origanum, cinnamon, origanum-clove-orange, and clove-lavender-cinnamon^62^.

The primary objectives of the current work were to first use GC–MS to identify the chemical components of the EO extracted from *Eucalyptus camaldulensis* leaves, and then to determine the quantum mechanical properties of the principal constituents of the EO. After treating four textiles, the EO and its two primary components were tested as antifungal agents against various fungal isolates that were identified from contaminated old textiles.

## Materials and methods

### Preparation of textile samples

This study has complied with relevant institutional, national, and international guidelines and legislation. This study does not contain any studies with human participants or animals performed by any of the authors. Four natural fabrics (linen, cotton, wool, and silk) were bought from Manstex Textile Company, Alexandria, Egypt; Misr Spinning & Weaving Company, Mahala El Kobra, Egypt; Goldentex Wool Textile Company, 10th of Ramadan, Egypt; and Awlad Khattab Company for Handmade Silk Textile, Akhmim, Egypt, respectively (Table 1).

**Table 1:** Characteristics of linen, cotton, wool, and silk samples.

Textile	Weave structure	Basic weight (g/m^2^)	Wefts (yarns/cm)	Warps (yarns/cm)	Textile strength (kg/strength)	Elongation (%)
Linen	Plain weave	113	13	17	56.5	6.9
Cotton	Plain weave	179	25	25	66.9	19.9
Wool	Plain weave	148	23	23	13.9	12.6
Silk	Plain weave	143	24	25	33.7	14.9

### Extraction of *Eucalyptus camaldulensis* essential Oil

The essential oil (EO) from leaves of *Eucalyptus camaldulensis* Dehnh. (Family Myrtaceae) were extracted by the hydrodistillation method using the Clevenger apparatus at the Faculty of Agriculture (EL-Shatby), Alexandria University, Alexandria, Egypt, where approximately 150 g of small pieces of leaves were put in a 2 L flask containing 1500 mL of distilled water and then connected to a Clevenger unit and heated for 3 h under refluxing^50,63^. The EO was collected and dried over anhydrous sodium sulfate and the EO amount was calculated based on sample weight^10^ as 2.44 mL/100 fresh leaves of *E. camaldulensis*. The collected EO was preserved at 4 °C in a refrigerator while being kept dry in an Eppendorf tube.

### GC–MS analysis of the essential oil from *E. camaldulensis*

The EO from *E. camaldulensis* were analyzed for their chemical composition using Focus GC-DSQ Mass Spectrometer (Thermo Scientific, Austin, TX, USA) with a direct capillary column TR–5MS (30 m × 0.25 mm internal diameter and 0.25 µm film thickness) apparatus at Atomic and Molecular Physics Unit, Experimental Nuclear Physics Department, Nuclear Research Centre, Egyptian Atomic Energy Authority, Inshas, Cairo, Egypt. The column oven temperature was initially held at 60 °C and then increased by 5 °C/min to 250 °C with holding 2 min and then increased to 300 °C (25 °C/min). Temperature of the injector were kept at 270 °C. Helium, the carrier gas, was kept in constant flow rate of 1 mL/min. The solvent delay was 4 min, and diluted samples of 1 µL were injected automatically using Auto-sampler AS3000 coupled with GC in the split mode. EI mass spectra were collected at 70 eV ionization voltages over the range of m/z 50–550 in full-scan mode. The ion source and transfer line temperatures were set at 200 and 250 °C, respectively. By comparing the components' retention times and mass spectra to those from the WILEY 09 and NIST 11 mass spectral databases, the components were identified.

A powerful method for analyzing EO or derivatized nonvolatile molecules at lower concentrations and providing a great depth of information is coupling chromatography to MS via gas chromatography-mass spectrometry (GC–MS). This method has higher sensitivity, selectivity, and structural determination capabilities than other structural characterization methods like IR and Raman spectroscopy^64^.

### Computation method

With the help of the molecular modeling program Hyperchem7.5 (W. Thiel 2003, HyperChemTM, Release 7.5 Pro 2002), the geometry of the compounds under study has been optimized on the basis of semi-empirical calculations^65^. Semi-empirical calculations were carried out using the routine Modified Neglect of Diatomic Overlap (MNDO) and the Polak–Ribiere conjugated gradient algorithm^66,67^. For the optimized structure at the ground state and reported the molecular electrostatic potential of the active functional groups of the studied components.

### Antifungal activity in vitro

**Figure 1:**
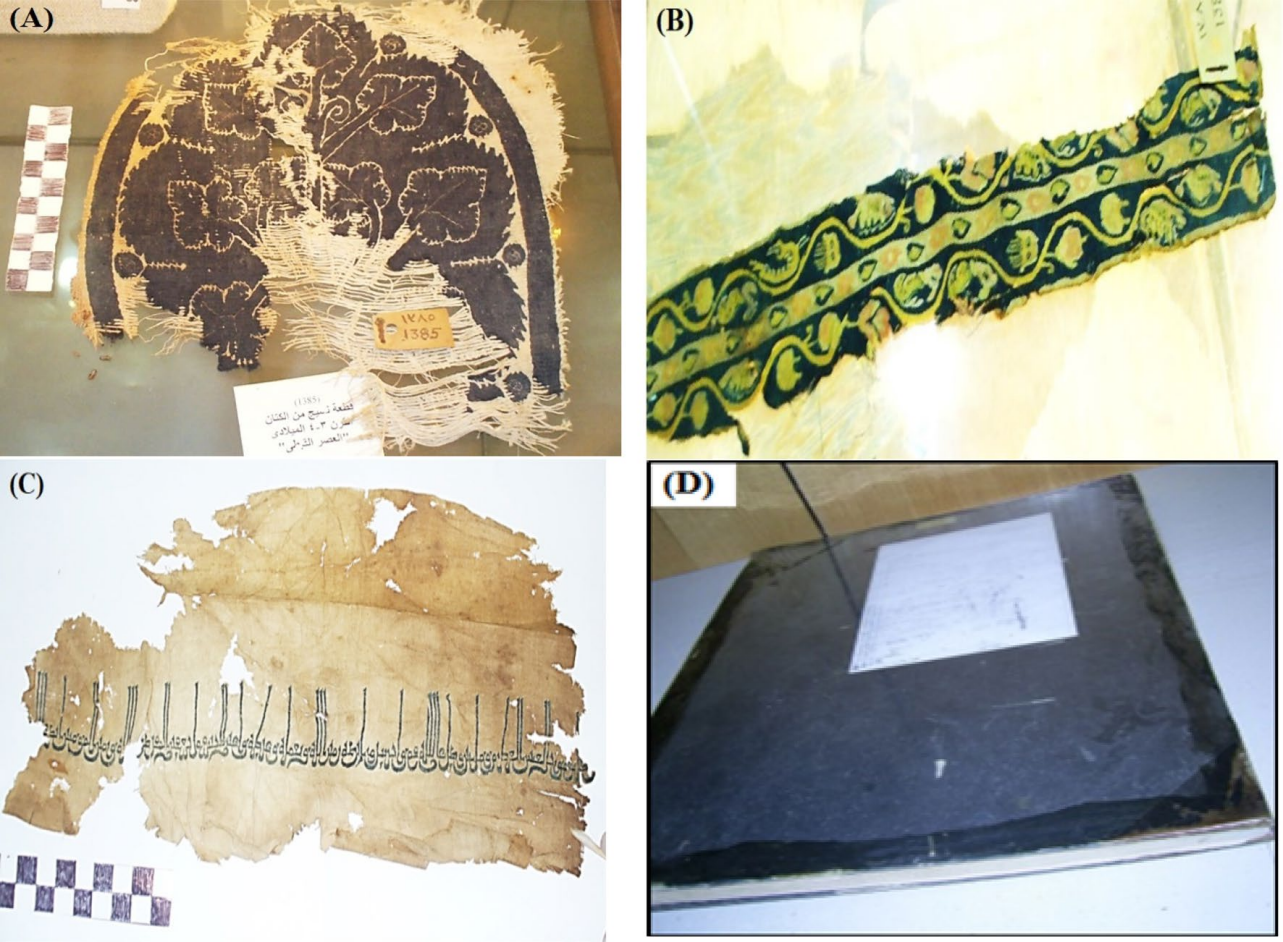
Contaminated ancient textiles. (**A**) Coptic textile No. 1385, a wool and linen item from the third or fourth century AD (26 × 24 cm) that was placed at the Faculty of Arts Museum, Alexandria University, (**B**) Woolen weaved No. 1381 from the Museum of the Faculty of Arts, Alexandria University, which dates to the sixth century AD, (**C**) Islamic woven inscription No. 1244, written in Kufic script, placed at Museum of the Faculty of Arts, Alexandria University (Photos were taken by coauthor Ibrahim H. M. Ibrahim), and (**D**) The antique textile from 1822 AD that was used to tie the Description of Egypt, which is housed in Cairo, Egypt's Central Library of Manuscripts (Photo was taken by coauthor Ayman S. Taha).

The antifungal bioassays of the EO and the main two compounds, eucalyptol and spathulenol, purchased from Sigma-Aldrich (Merck), were conducted using three molds (*Aspergillus flavus* AFl375, *Fusarium culmorum* Fcu761, and *Aspergillus niger* Ani245) and accession numbers in Gen Bank, MH355958, MH355955, and MH355957, respectively^68,69^. These fungal strains were previously isolated from contaminated ancient textiles (Fig. 1A–D)^70^. The extracted EO from *E. camaldulensis* leaves was prepared at the concentrations of 125, 250, and 500 µL/L, while eucalyptol and spathulenol were prepared at 6, 12, 25, and 50 µL/L. Tween 40 (0.5 mL) was added after the EO had been diluted in 10% DMSO, in order to disseminate the EO more widely and ensure homogeneous evaporation.

Samples of linen, cotton, wool, and silk in the dimensions of 2 × 2 cm were exposed to the prepared EO and eucalyptol and spathulenol using the vapor method^71,72^. The treated and un-treated textiles were exposed to the fungal infestation using the three molds. The samples were inoculated with a 9-mm disc diameter from each fungus' 15-day-old PDA culture in Petri dishes and incubated for 14 days at 25 ± 1°C. Following inoculation, the growth on disc (mm) and inhibition zone (mm) for each fungus were measured 7 and 14 days after inoculation for textiles treated with EO and after 14 days for textiles treated with eucalyptol and spathulenol^70,73^.

The eucalyptus oil's minimal inhibitory concentrations (MICs) were examined at concentrations between 7.5 and 500 µL/L, while those for the components eucalyptol and spathulenol were studied from 1 to 50 µL/L^50,74^.

### Scanning electron microscopy

Fungal infestations over the four the samples textile were examined using a Scanning Electron Microscope. The samples were coated with gold in a fine coat and examined via SEM-JEOL (JFC-1100E Ion sputtering device, model JSM- 5300, JEOL Co., Tokyo, Japan) at 8 Kv^26^.

### Statistical analysis

The effects of the EO concentrations or the main two compounds and the treated textiles with the EO as well as their interactions on the inhibition zone values and the growth of the tested fungi over the textile samples were statistically analyzed in a two-way ANOVA analysis of variance (ANOVA) using SAS software (SAS Institute, Release 8.02, Cary, North Carolina State University, Raleigh, NC, USA), and the means were compared against the control treatment. Additionally, ANOVA with three factors was used to study the significant effects of monoterpenes (eucalyptol and spathulenol) with their concentrations when applied to the four textiles on the IZ and the growth on textile samples after 14 days incubation with *A. flavus*, *F. culmorum*, and *A. niger*.

## Results and discussion

### Fibers identification

Figure S1 displays the identification of the investigated fibers. Longitudinal views of fibers are more distinctive and special, where linen thread has thick nodes with crossings and the typical polygonal structure of the fibers (Fig. S1a,b), whereas wool fiber has scales that are, for example, plainly visible even when covered by a thin coating of dirt (Fig. S1c)^75–77^. The cotton fibers seem like a twisted ribbon (Fig. S1d,e), compared to silk fibers longitudinal views are straight (Fig. S1f)^78–80^.

### Chemical composition of *E. camaldulensis* essential oil

Figure S2 depicts the determined chemical composition of the EO from *E. camaldulensis*, and Table 2 lists 26 components. The most abundant compounds were spathulenol (26.56%), eucalyptol (14.91%), *p*-cymene (12.40%), crypton (7.72%), carvacrol (4.21%), phellandral (4.55%), alloaromadendrene oxide-(2) (3.22%), 4-terpineol (3.00%), cuminaldehyde (2.97%), ylangenal (2.89%), aromandendrene (2.79%), *p*-cymen-7-ol (1.73%), 4(15),5,10(14)-germacratrien-1-ol (1.49%), and ledol (1.22%).

**Table 2:** GC–MS analysis of the phytochemicals in *E. camaldulensis* leaves' essential oil.

RT (min)	Compound	Percentage (%)	Molecular weight	Molecular formula	RI*	MF**	Most intense ions %
4.55	(-)-beta-Pinene	0.31	136	C_10_H_16_	979	915	136(4%), 121(12%), 93(100%), 79(20%) and 69(72%)
5.27	p-Cymene	12.40	134	C_10_H_14_	1025	948	134(38%), 119(100%) and 91(25%)
5.44	Eucalyptol	14.91	154	C_10_H_18_O	1032	921	154(42%), 139(58%), 108(63%), 81(100%) and 81(85%)
7.55	trans-para-2-menthen-1-ol	0.71	154	C_10_H_18_O	1140	909	154(20%), 139(85%), 121(82%), 93(89%) and 79(100%)
7.98	3-Terpinen-1-ol	0.58	154	C_10_H_18_O	1158	902	154(18%), 139(100%), 121(89%), 93(80%) and 69(81%)
8.94	4-Terpineol	3.00	154	C_10_H_18_O	1137	940	154(21%), 136(15%), 111(62%) , 93(65%) and 71(100%)
9.07	Crypton	7.72	138	C_9_H_14_O	1184	948	138(20%), 96(95%), 95(100%) , 81(25%) and 67(42%)
10.50	Cuminaldehyde	2.97	148	C_10_H_12_O	1239	949	148(78%), 133(100%), 105(82%) and 77(42%)
11.39	Phellandral	4.55	152	C_10_H_16_O	1276	944	152(10%), 109(100%), 95(38%), 81(42%) and 67(38%)
11.82	*p*-Cymen-7-ol	1.73	150	C_10_H_14_O	1289	939	150(45%), 135(100%), 105(58%) and 79(43%)
12.07	Carvacrol	4.21	150	C_10_H_14_O	1299	929	150(41%), 135(100%), 107(19%) and 91(20%)
12.55	2,3-Pinanediol	0.97	170	C_10_H_18_O_2_	1244	769	170(5%), 126(80%), 111(100%), 93(40%) and 71(58%)
15.95	Aromandendrene	2.79	204	C_15_H_24_	1440	958	204(25%), 189(23%), 161(80%), 133(78%),105(98%), 91(100%) and 79(65%)
16.64	Aromadendrene, dehydro-	1.18	202	C_15_H_22_	1464	715	202(3%), 157(60%), 145(100%),105(40%), 91(25%) and 79(15%)
18.28	Guaiene	0.58	204	C_15_H_24_	1490	829	204(48%), 189(43%), 161(65%), 133(75%),105(96%), 91(100%) and 79(45%)
18.77	Spathulenol	26.56	220	C_15_H_24_O	1576	951	220(10%), 205(85%), 159(80%), 147(59%), 119(85%, 91(100%) and 79(60%)
18.92	Alloaromadendrene oxide-(2)	3.22	220	C_15_H_24_O	1462	837	220(2%), 205(15%), 187(20%), 161(59%), 107(100%, 81(92%) and 79(80%)
19.25	trans-Longipinocarveol	0.72	220	C_15_H_24_O	1618	800	220(5%), 187(25%), 159(60%), 132(100%, 120(79%), 105(65%) and 91(61%)
19.35	Ledol	1.22	222	C_15_H_26_O	1565	918	222(3%), 189(21%), 161(62%), 133(45%, 122(83%), 107(100%), 81(83%) and 67(68%)
20.01	Isospathulenol	0.77	220	C_15_H_24_O	1638	902	220(16%), 187(23%), 162(58%), 147(61%, 119(100%), 191(85%) and 58(42%)
20.30	α-Vetivol	0.59	220	C_15_H_24_O	1756	838	220(40%), 187(58%), 160(100%), 131(78%, 105(65%), 91(62%) and 58(69%)
20.50	ent-Germacra 4(15),5,10(14)-trien-1β-ol	1.49	220	C_15_H_24_O	1695	848	220(10%), 205(45%), 159(100%), 121(79%, 105(98%), 91(97%) and 58(40%)
20.70	Ledene oxide-(II)	0.74	220	C_15_H_24_O	1631	831	220(18%), 187(15%), 177(100%), 159(80%, 123(82%), 91(47%) and 58(25%)
20.92	Caryophyllene oxide	0.57	220	C_15_H_24_O	1690	826	220(10%), 187(42%), 159(45%, 119(58%), 107(100%) and 79(60%)
22.14	Ylangenal	2.89	218	C_15_H_22_O	1675	871	218(20%), 189(42%), 175(94%), 105(83%), 91(100%) and 79(78%)
22.59	β-Longipinene	1.14	204	C_15_H_24_	1403	855	204(2%), 175(39%), 133(80%), 105(83%), 91(100%) and 79(65%)
25.46	Verrucarol	0.56	266	C_15_H_22_O_4_	1939	807	266(1%), 219(21%), 191(18%, 149(45%), 121(58%), 107(80%), 91(100%) and 79(85%)
Total percentage (%)		99.08					

*R.I.: retention index (All analysis of studied samples have been done using Thermo fisher GCMS which supported by number of mass spectral libraries and all separated compounds haven been identified and all data have been reported using NIST014 which already supported by retention index library (RI) for 82,868 compounds (https://www.nist.gov/system/files/documents/srd/NIST1aVer22Man.pdf).

**MF: match factor.

The molecular mass, chemical formula, chemical structure, or quantity of the analyte can be determined using mass spectrometric analysis of the organic components found in the EO made from *E. camaldulensis* leaves (Table 2). The formula and chemical structure can be ascertained manually^81^ and/or by comparison with a reference database of spectra based on the measured m/z and their peak intensities.

In the high-throughput examination of chemicals synthesized in combinatorial libraries, such as those created to find new medicines, mass spectra (MS) are also a useful technique. Both structural data and information regarding a molecule's binding affinity can be collected if the mass spectrometer is coupled to an LC system with the right columns^82^. By listing the molecular and fragment ions of a chemical, it was demonstrated that the mass spectrum is actually nothing more than a depiction of the substance's molecular structure. Any molecule's structure determines its pharmacological and biological activity, and a change in structure might result in a change in activity^83,84^.

**Figure 2:**
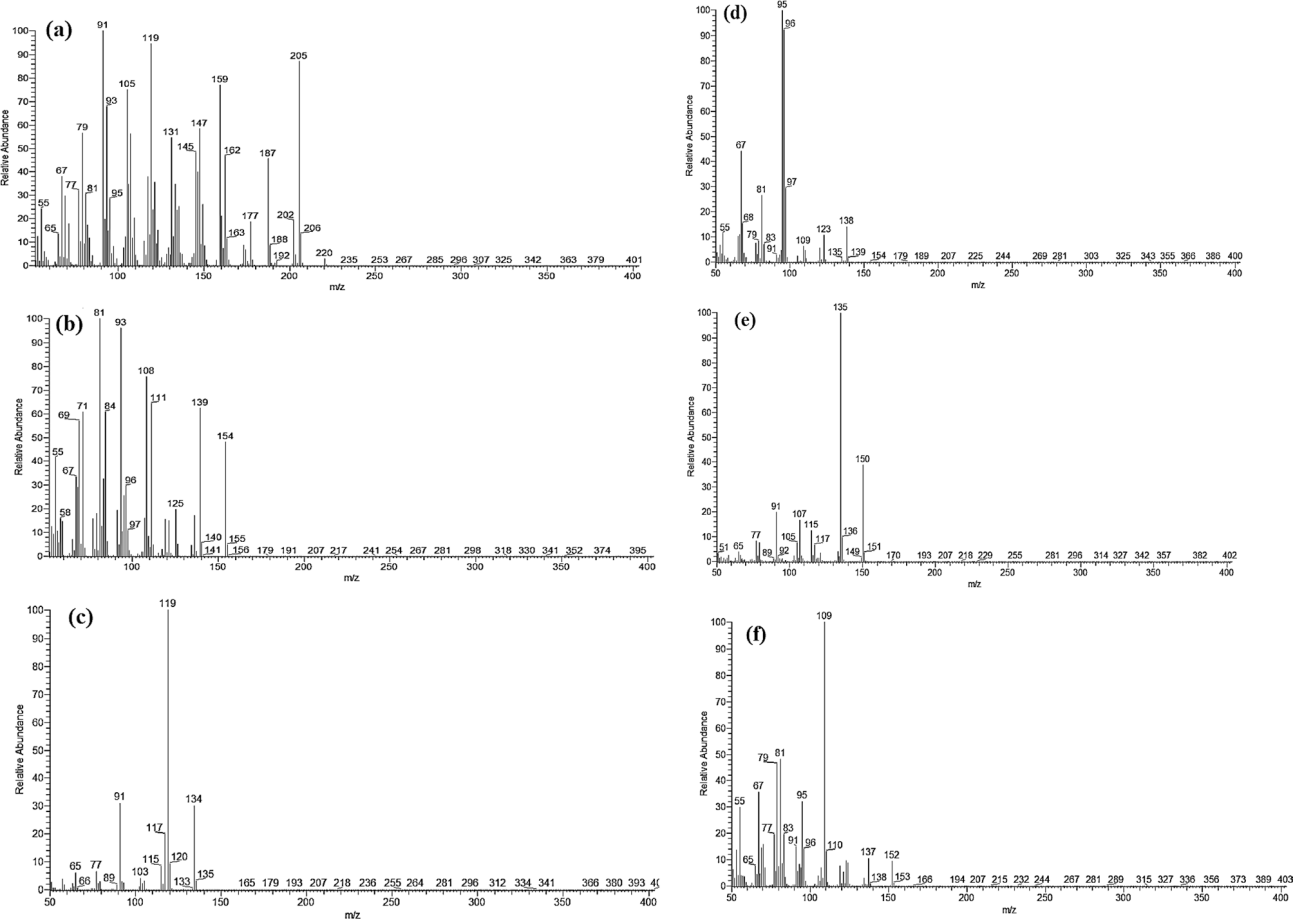
The 70 eV mass spectrum of (**a**) spathulenol component at RT 18.77 min, (**b**) Eucalyptol component at RT 5.44 min, (**c**) *p*-cymene component at RT 5.27 min, (**d**) Crypton component at RT 9.07 min, (**e**) carvacrol component at RT 12.07 min, and (f) Phellandral component at RT 11.39 min.

The principle EO components rom *E. camaldulensis* had their 70 eV MS recorded and explained, as illustrated in Fig. 2a–f. The spathulenol component is represented by the peak at retention time (RT) of 18.77 min (Fig. 2a), which suggests that its chemical formula is C_15_H_24_O (Table 2). The molecular ion peak was detected at m/z 220 with a relative intensity (RIn) of 10%, while and the base peak is represented by the peak at 91 (RIn = 100%). Significant fragment ions were also found at positions 205(85%), 159(80%), 147(59%), 119(85%), and 79(60%).

The peak at RT 5.44 min (Fig. 2b) reveals the eucalyptol component, which is indicated by the MS and has the chemical formula C_10_H_18_O. A persistent molecular ion peak was found at m/z 154 with RIn = 42%, and the base peak is found at m/z 81 with RIn = 100%. The following notable fragment ions were also found: 139 (58%), 108 (63%), and 81 (85%). The p-cymene component is seen in the MS of the peak at RT 5.27 min (Fig. 2c), which proves its chemical formula is C_10_H_14_ (Table 2). The base peak, which is represented by the peak at 119 (RIn = 100%), is the molecular ion peak, which was seen at m/z 134 with RIn = 38% (stable molecular ion). The detected fragment ion of 91 (25%) was also noteworthy.

The crypton component is represented by the MS of the peak at RT 9.07 min (Fig. 2d), indicating that its chemical formula is C_9_H_14_O (Table 2). A persistent molecular ion peak was seen at m/z 138 with RIn = 20%, while the base peak was seen at m/z 95 with RIn = 100%. Fragment ions at 96 (95%), 81 (25%), and 67 (42%), where the other notable ones that were found. The carvacrol component is represented by the peak at RT 12.07 min (Fig. 2e), which suggests that its chemical formula is C_10_H_14_O (Table 2). The base peak is represented by the peak at 135 (RIn = 100%), while the molecular ion peak was found at m/z 150 with RIn = 41% (stable molecular ion). Other major fragment ions of 107 (19%), and 91 (20%) were observed.

The phellandral component is represented by the peak at RT 11.39 min (Fig. 2f), which suggests that it has the chemical formula C_10_H_16_O (Table 2). The molecular ion peak was detected at m/z 152 with RIn = 10%, while the base peak is represented by the peak at 109 (RIn = 100%). There were also notable fragment ions of 95(38%), 81(42%), and 67(38%).

### Molecular electrostatic potential (MEP)

**Figure 3:**
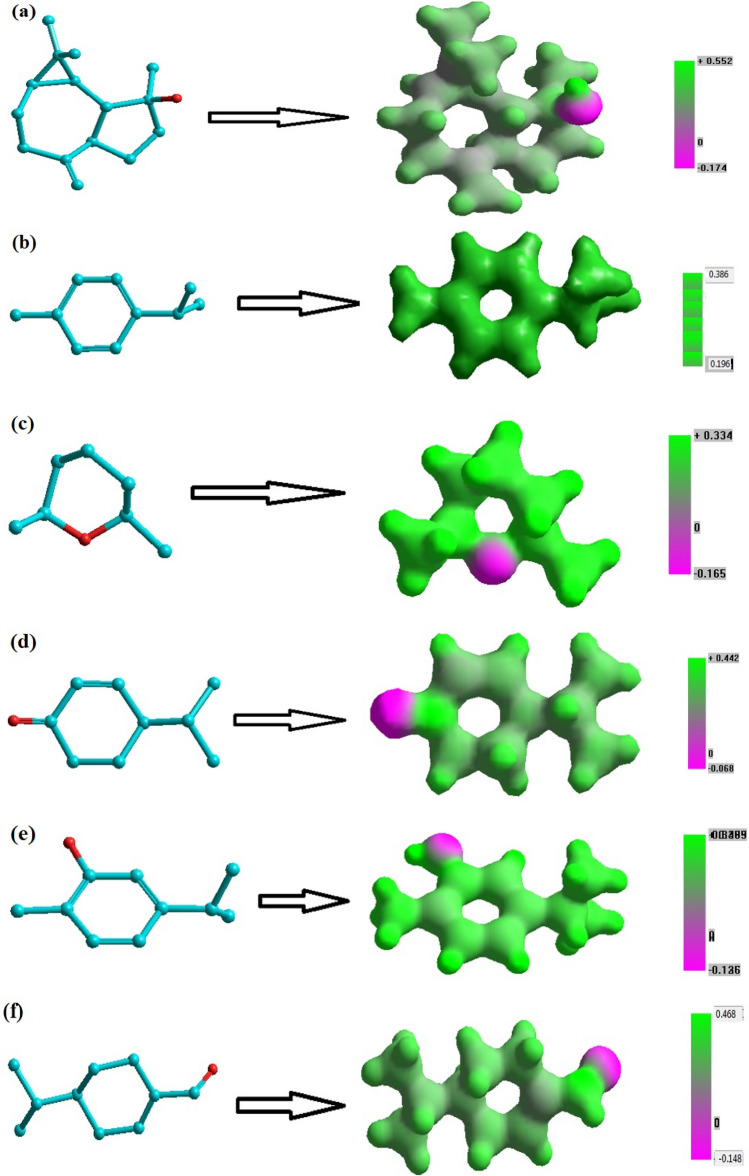
The optimized structures and electrostatic potential 3D mapped using the HyperChem program using MNDO method for the studied components. (**a**) Spathulenol, (**b**) p-Cymene, (**c**) Eucalyptol, (**d**) Crypton, (**e**) Carvacrol; and (**f**) Phellandral.

Figure 3a–f displays the MEP plots for the primary chemicals. With the exception of the *p*-cymene molecule (Fig. 3b), it is possible to see that the investigated molecules' regions surrounding the oxygen atoms linked to the heterocyclic ring are the most electrophilic site (red regions), as a result of the concentrated electron density. Additionally, spathulenol (Fig. 3a) has the highest electrostatic potential (ESP) compared to the other key *E. camaldulensis* EO compounds, which explains why it makes up the majority of this molecule in the chemical composition in the EO extracted from *E. camaldulensis* leaves. On the other hand, the nucleophilic site of a *p*-cymene molecule is represented by the areas close to the carbon atom (green regions).

Semi-empirical calculations were carried out using the routine MNDO and Polak–Ribiere conjugated gradient algorithm and the program MNDO were used to do semi-empirical computations. The molecular charge distribution is unaffected by the external test charge, making molecular electrostatic potential (MEP) a useful tool for determining the reactive sites toward positively or negatively charged reactants and enabling the establishment of the hydrogen bonding and structure–activity relationships of the molecule^85^. Quantum chemical calculations have shown that the electrostatic potential, electronegativity, partial charges, and dipole moment are strongly correlated^86^. By plotting the total density surface on the electrostatic potential energy surface and showing the size, shape, charge density, and reactive sites of the molecules, MEP offers a visual way to comprehend the relative polarity of a molecule.

### Antifungal activity

After 7, and 14 days of incubation, Table S1 displays the significant impacts of EO concentrations, the types of textiles treated with the EO, and their interactions on the inhibition zones (IZs) and the growth of *Aspergillus flavus*, *Fusarium culmorum*, and *A. niger*. As can be seen, the ANOVA test revealed significant differences between the level concentrations of *E. camaldulensis* EO, the types of textiles treated with the EO, and their interactions, with the exception of the interaction between EO concentrations and treatments on the growth of *A. flavus* on samples after 14 days of incubation (*p* = 0.5093) and the impact of textiles treated with the EO on the IZs after 7 days of incubation against *F. culmorum* (*p* = 0.0879).

Table 3 and Fig. S3 show the IZs (mm) and the growth on the textile samples treated with EO after 7 and 14 days of *A. flavus* incubation. The treated Silk, Linen, and Cotton fabrics showed the highest IZs at 500 μL/L after 7 days of incubation, with values of 8.33, 5.66, and 4 mm, respectively. These values fell to 7, 4.33, and 3.33 mm, respectively, after 14 days of incubation. While wool materials had a 0.33 mm growth after 14 days of *A. flavus* incubation, treated cotton, linen, and silk with 500 µL/L of *E. camaldulensis* EO did not exhibit any visible growths. After 14 days of incubation and in the control treatments, the growth over the textiles made of cotton, linen, silk and wool was 20, 19.66, 19.66, and 18 mm, respectively.

**Table 3:** Antifungal activity of *E. camaldulensis* EO-treated textiles against the growth of *Aspergillus flavus.*

Textile fabrics	EO concentration (μL/L)	Inhibition zone (mm)		Growth on sample (mm)	
		7th day	14th day	7th day	14th day
Cotton	125	0.00	0.00	3.33 ± 1.15	12 ± 2.64
Cotton	250	0.33 ± 0.57	0.00	1 ± 1	3.66 ± 1.52
Cotton	500	4 ± 1	3.33 ± 0.57	0.00	0.00
Cotton	Control	0.00	0.00	12.33 ± 2.51	20
Linen	125	0.00	0.00	4.33 ± 1.15	11.33 ± 1.15
Linen	250	0.66 ± 1.15	0.00	1.33 ± 1.15	3 ± 1
Linen	500	5.66 ± 0.57	4.33 ± 0.57	0.00	0.00
Linen	Control	0.00	0.00	9 ± 1	19.66 ± 0.57
Silk	125	0.66 ± 0.57	0.00	1 ± 1	10.33 ± 1.52
Silk	250	1.33 ± 1.15	0.33 ± 0.57	0.33 ± 0.57	1.66 ± 1.52
Silk	500	8.33 ± 1.52	7 ± 1	0.00	0.00
Silk	Control	0.00	0.00	10.66 ± 1.15	19.66 ± 0.57
Wool	125	0.00	0.00	1.33 ± 0.57	9 ± 1
Wool	250	0.66 ± 1.15	0.33 ± 0.57	0.00	2.33 ± 2.08
Wool	500	1 ± 1	0.66 ± 0.57	0.00	0.33 ± 0.57
Wool	Control	0.00	0.00	9 ± 1	18 ± 2
*P-value*		< 0.0001	< 0.0001	0.0047	0.5093

After 7 and 14 days of incubation, Table 4 and Fig. S4 demonstrate the *E. camaldulensis* EO’s bioactivity against the development of *F. culmorum*. The maximum IZ values of 6, 4.33, 3.66, and 3.33 mm against the growth of *F. culmorum* were seen after 7 days of incubation, when silk, linen, cotton and wool samples, respectively, were treated with 500 µL/L of EO concentration. However, after 14 days of incubation, these values fell to 5, 4, 3.33, and 2.66 mm, respectively.

**Table 4:** Antifungal activity of *Eucalyptus camaldulensis* EO-treated textiles against the growth of *Fusarium culmorum.*

Textile fabrics	EO concentration (μL/L)	Inhibition zone (mm)		Growth on sample (mm)	
		7th day	14th day	7th day	14th day
Cotton	Control	0.00	0.00	19 ± 1	19 ± 2
Cotton	125	0.00	0.00	19.66 ± 0.57	20
Cotton	250	1 ± 1	0.00	0.33 ± 0.57	1
Cotton	500	3.66 ± 0.57	3.33 ± 0.57	0.00	0
Linen	Control	0.00	0.00	19.33 ± 0.57	20
Linen	125	0.00	0.00	19.33 ± 0.57	20
Linen	250	0.66 ± 0.57	0.00	0.33 ± 0.57	1.33 ± 0.57
Linen	500	4.33 ± 1.15	4 ± 1	0.00	0.00
Silk	Control	0.00	0.00	19.33 ± 1.52	20
Silk	125	0.00	0.00	19.33 ± 0.57	20
Silk	250	0.00	0.00	1.33 ± 1.15	2.33 ± 0.57
Silk	500	6 ± 1	5 ± 1	0.00	0.00
Wool	Control	0.00	0.00	19.66 ± 4.04	20
Wool	125	0.00	0.00	19.33 ± 0.57	20
Wool	250	0.33 ± 0.57	0.00	0.66 ± 0.57	1.66 ± 0.57
Wool	500	3.33 ± 0.57	2.66 ± 0.57	0.00	0.00
*p*-value		0.0005	0.0008	< 0.0001	0.0244

After 7 days from inoculation, *F. culmorum* fungal growth ranged from 19 to 19.66 mm in cotton, linen, silk, and wool in the control treatment, and from 19.66 to 19.33 mm in samples treated with 125 µL/L of *E. camaldulensis* EO. These values roughly reached 20 mm after 14 days of incubation. After 7 or 14 days of incubation, no fungus was discovered over the textile samples that had been treated with 500 µL/L of *E. camaldulensis* EO.

The antifungal activity of the textiles treated with *E. camaldulens* EO against the growth of *A. niger* is shown in Table 5 and Fig. S5.The treated cotton, linen, silk and wool with 500 µL/L of *E. camaldulensis* EO revealed IZ values of 8.66, 6.33, 6, and 5.33 mm, respectively, after 7 days of incubation with *A. niger*. After 14 weeks of incubation, these values marginally dropped to 6.66, 4.66, 5.33, and 3.66 mm, respectively. After 14 days of incubation, the fungal growth in untreated cotton, linen, silk, and wool reached 19.66, 20, 20, and 19.33 mm, respectively. This growth was halted in textiles treated with 500 µL/L of *E. camaldulensis* EO.

**Table 5:** Antifungal activity of *Eucalyptus camaldulensi* EO-treated textiles against the growth of *Aspergillus niger.*

Textile fabrics	EO concentration (μL/L)	Inhibition zone (mm)		Growth on sample (mm)	
		7th day	14th day	7th day	14th day
Cotton	Control	0.00	0.00	16.66 ± 0.57	19.66 ± 0.57
Cotton	125	0.00	0.00	11.33 ± 1.15	19.66 ± 0.57
Cotton	250	0.00	0.00	12.33 ± 2.512	17.33 ± 1.15
Cotton	500	8.66 ± 0.57	6.66 ± 1.15	0.00	0.00
Linen	Control	0.00	0.00	17.66 ± 0.57	20
Linen	125	0.00	0.00	12.33 ± 2.51	19.33 ± 1.15
Linen	250	1.66 ± 0.57	0.00	0.00	3.33 ± 0.57
Linen	500	6.33 ± 0.57	4.66 ± 0.57	0.00	0.00
Silk	Control	0.00	0.00	14.33 ± 0.57	20
Silk	125	0.00	0.00	12.33 ± 3.21	16.66 ± 0.57
Silk	250	0.33 ± 0.57	0.00	1.66 ± 1.52	10.66 ± 1.52
Silk	500	6 ± 1	5.33 ± 0.57	0.00	0.00
Wool	Control	0.00	0.00	11.66 ± 3.51	19.33 ± 1.15
Wool	125	0.00	0.00	5.66 ± 1.15	7.66 ± 1.15
Wool	250	0.00	0.00	2.33 ± 2.08	4 ± 2.64
Wool	500	5.33 ± 0.57	3.66 ± 0.57	0.00	0.00
*p-value*		< 0.0001	< 0.0001	< 0.0001	< 0.0001

### SEM microscopy observation

**Figure 4:**
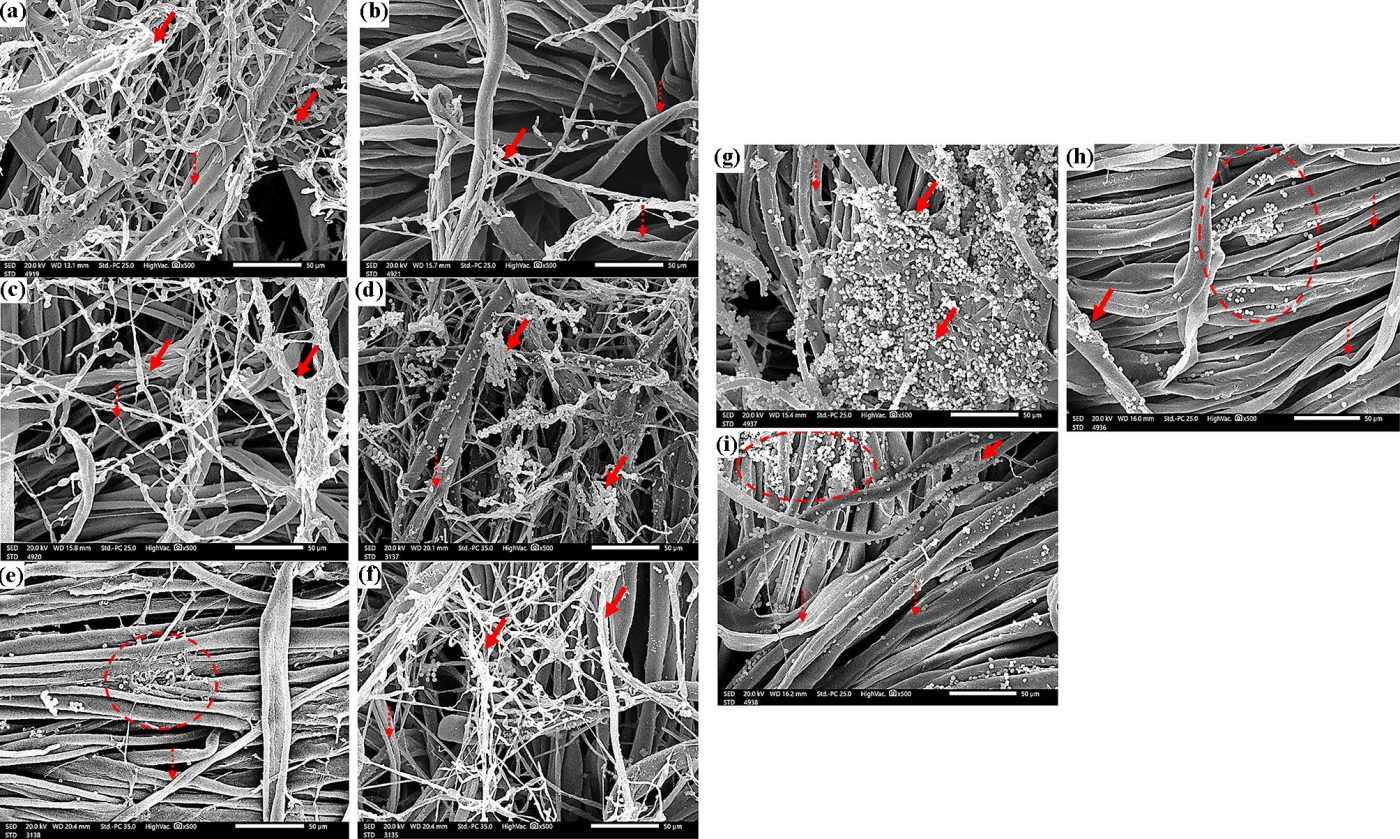
SEM images of cotton samples inoculated with: *A. flavus* without treatment (**a**), *A. flavus* with 250 µL/L of *E. camaldulensis* EO (**b**), *A. flavus* with 125 µL/L of *E. camaldulensis* EO (**c**), *F. culmorum* without treatment (**d**), *F. culmorum* with 250 µL/L of *E. camaldulensis* EO (**e**), *A. flavus* with 125 µL/L of *E. camaldulensis* EO, (**f**), *A. niger* without treatment (**g**), *A. niger* with 250 µL/L of *E. camaldulensis* EO (**h**), and with 125µL/L of *E. camaldulensis* EO (**i**). The growth of fungal mycelia dependent on EO treatment concentrations is indicated by solid arrows, while the longitudinal twists of cotton fiber are indicated by dotted arrows.

The SEM was used to compare the patterns of fungal development in the treated and untreated textiles by *E. camaldulensis* EO inside and around the fibers. *A. flavus* showed extensive growth in cotton samples that had not been treated with EO (Fig. 4a); this growth nay have deceased in cotton samples that had been treated with 250 µL/L of *E. camaldulensis* EO (Fig. 4b); however the mycelial net of *A. flavus* was still visible in cotton samples that had been treated with 125 µL/L of *E. camaldulensis* EO (Fig. 4c).

The same trend was observed under SEM examination, where a huge growth of *F. culmorum* (Fig. 4d) and *A. niger* (Fig. 4g) was found in untreated cotton samples and these growths were significantly decreased as the samples treated with 250 µL/L of *E. camaldulensis* EO (Fig. 4e,h) with some growths were shown in samples treated with 125 µL/L *E. camaldulensis* EO (Fig. 4f,i).

**Figure 5:**
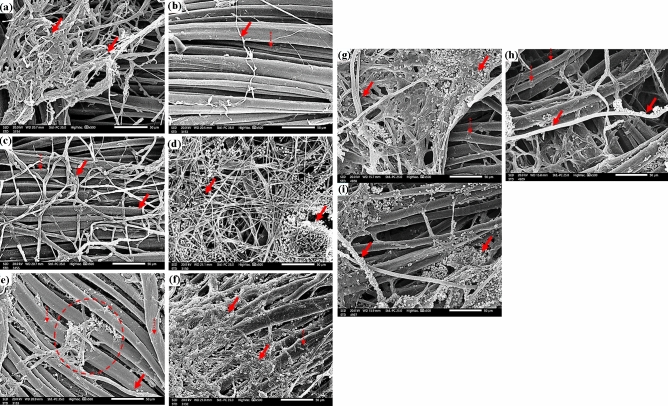
SEM images of linen samples inoculated with *A. flavus* without treatment (**a**), *A. flavus* with 250 µL/L of *E. camaldulensis* EO (**b**), *A. flavus* with 125 µL/L of *E. camaldulensis* EO (**c**), *F. culmorum* without treatment (**d**) *F. culmorum* with 250 µL/L of *E. camaldulensis* EO (**e**), *F. culmorum* with 125 µL/L of *E. camaldulensis* EO (**f**), *A. niger* without treatment (**g**), *A. niger* 250 µL/L of *E. camaldulensis* EO (**h**), and *A. niger* with 125 µL/L of *E. camaldulensis* EO (**i**). Solid arrows point to the development of fungus mycelia in response to EO concentrations, whereas dot-filled arrows depict the distinctive nodes of flax fibers in a longitudinal view.

Without any treatments, fungal mycelial grew rapidly on linen samples infected with *A. flavus* (Fig. 5a), *F. culmorum* (Fig. 5d), and *A. niger* (Fig. 5g). When the linen samples were treated with 250 µL/L of *E. camaldulensis* EO (Fig. 5b,e,h), the fungal growth was obviously suppressed, whereas some growth was prevented when the linen samples were treated with 125 µL of *E. camaldulensis* EO (Fig. 5c,f,i).

**Figure 6:**
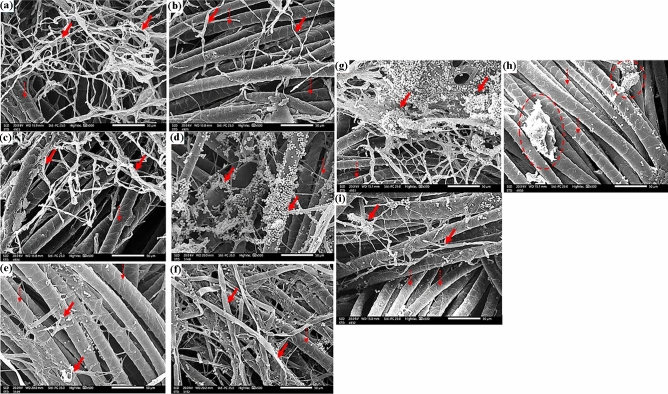
SEM images of wool samples inoculated with *A. flavus* without treatment (**a**), *A. flavus* with 250 µL/L of *E. camaldulensis* EO (**b**), *A. flavus* with 125 µL/L of *E. camaldulensis* EO (**c**), *F. culmorum* without treatment (**d**); *F. culmorum* with 250 µL/L of *E. camaldulensis* EO (**e**), *F. culmorum* with 125 µL/L of *E. camaldulensis* EO (**f**), *A. niger* without treatment (**g**), *A. niger* 250 µL/L of *E. camaldulensis* EO (**h**), and *A. niger* with 125 µL/L of *E. camaldulensis* EO (**i**). The growth of fungus-based mycelia is indicated by solid arrows, while the distinct nods of wool fiber's longitudinal perspective are indicated by dotted arrows.

*A. flavus* growth on untreated wool samples can be seen under a microscope (Fig. 6a), and growth on wool samples treated with 250 µL/L of *E. camaldulensis* EO dramatically decreased with destruction in the mycelial structures (Fig. 6b). The amount of fungal development that was reduced in the treated wool with 125 µL/L of *E. camaldulensis* EO was not noticeably greater (Fig. 6c).

Similar to how *F. culmorum* colonies were seen on wool samples without treatment (Fig. 6d), they significantly decreased after being treated with 250 µL/L of *E. camaldulensis* EO (Fig. 6e), and *F. culmorum* hyphae were visible on wool samples treated with 125 µL/L of *E. camaldulensis* EO (Fig. 6f). The heavy and intensive growth of *A. niger* on the untreated wool samples is clearly visible in Fig. 6g, while the wool treated with 250 µL/L of *E. camaldulensis* EO (Fig. 6h) or 125 µL/L of *E. camaldulensis* EO (Fig. 6i) showed a reduction in conidia.

**Figure 7:**
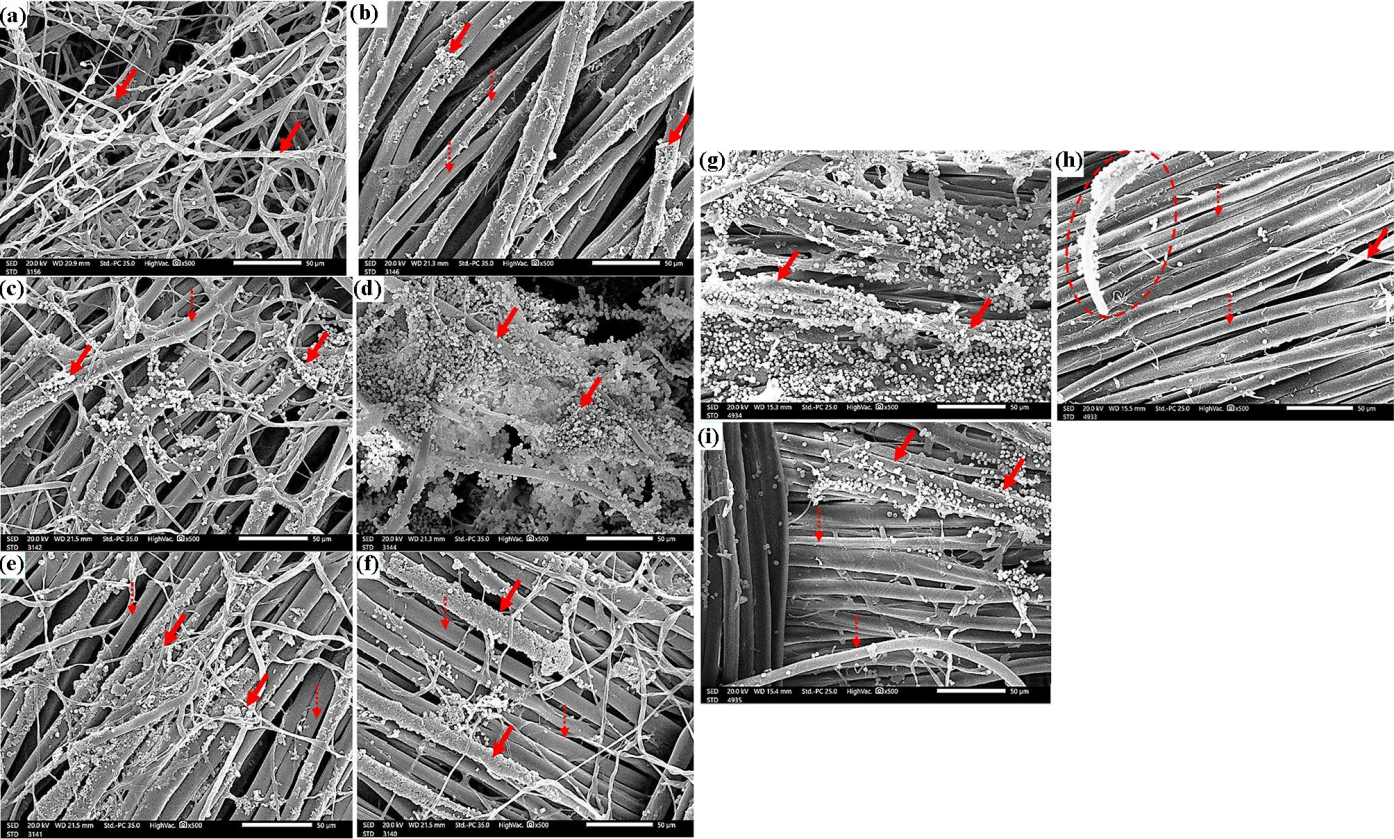
SEM images of silk samples inoculated with *A. flavus* without treatment (**a**), *A. flavus* with 250 µL/L of *E. camaldulensis* EO (**b**), *A. flavus* with 125 µL/L of *E. camaldulensis* EO (**c**), *F. culmorum* without treatment (**d**); *F. culmorum* with 250 µL/L of *E. camaldulensis* EO (**e**), *F. culmorum* with 125 µL/L of *E. camaldulensis* EO (**f**), *A. niger* without treatment (**g**), *A. niger* 250 µL/L of *E. camaldulensis* EO (**h**), and *A. niger* with 125 µL/L of *E. camaldulensis* EO (**i**). The growth of fungus mycelia based on EO concentration treatments is indicated by solid arrows; the longitudinal view of silk fibers is indicated by dotted arrows.

Figure 7 compares the silk textile that had been previously treated with *E. camaldulensis* EO and had been inoculated with *A. flavus*, *F. culmorum*, and *A. niger* to the untreated material. The untreated silk samples showed massive expansion of the mycelial net of *A. flavus* (Fig. 7a), *F. culmorum* (Fig. 7d), and *A. niger* (Fig. 7g). As the silk samples treated with 250 µL/L of *E. camaldulensis* EO against *A. flavus* (Fig. 7b), *F. culmorum* (Fig. 7e) and *A. niger* (Fig. 7h), significant decreases in this net of fungal hyphae were seen. Silk samples treated with 125 µL/L of *E. camaldulensis* EO and inoculated with *A. flavus*, *F. culmorum*, and A. *niger* (Fig. 7c, h, and i, respectively) revealed some reduction in the fungal growth as well.

### Antifungal activity of eucalyptol and spathulenol

**Figure 8:**
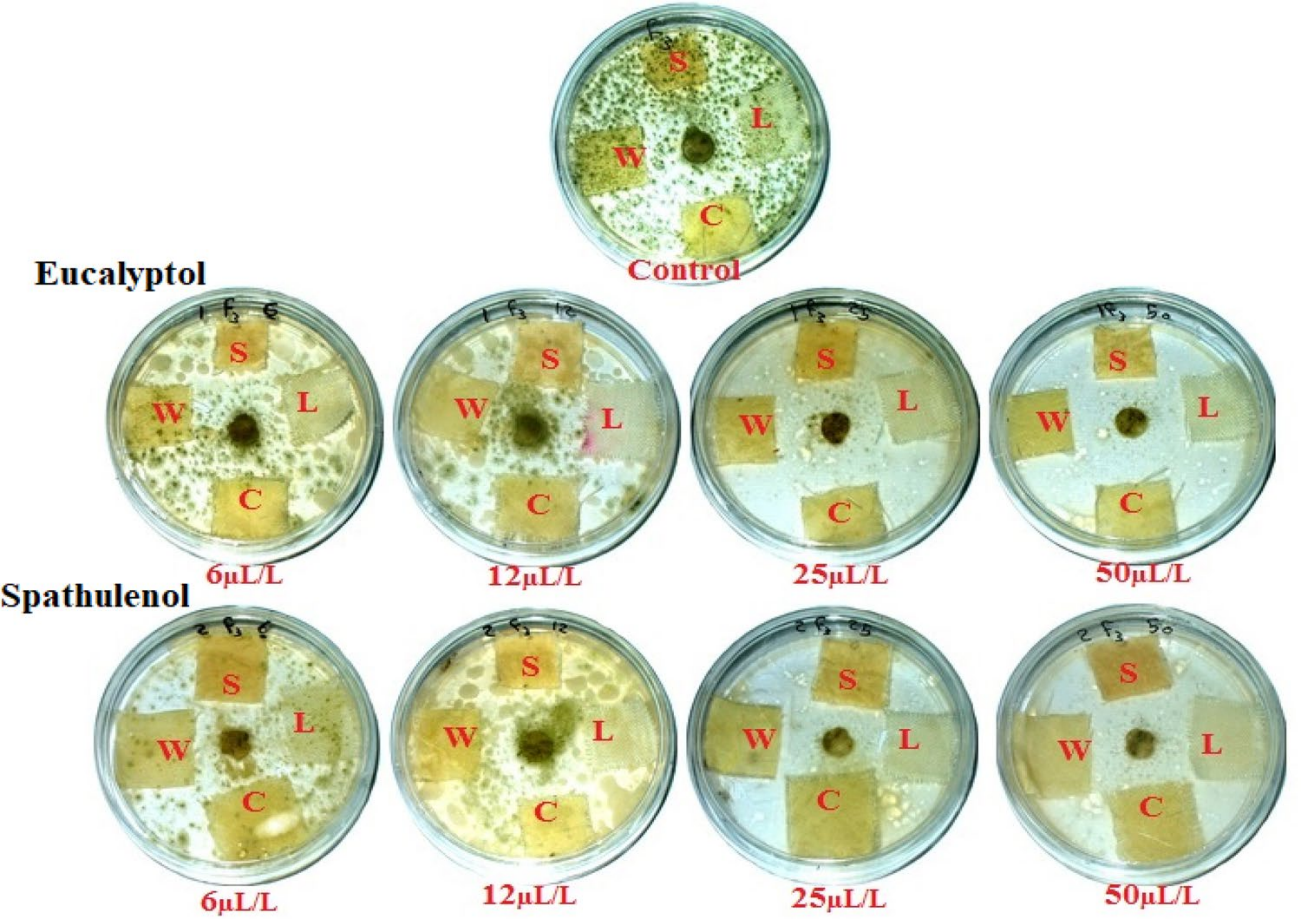
Visual observation of the effects of eucalyptol and spathulenol when treated textiles against the growth of *A. flavus*. (Photos were taken by coauthor Wael A. A. Abo-Elgat). S: Silk; W: Wool; L: Linen; and C: Cotton.

Table S2 demonstrate the significant effects of monoterpenes’ concentrations, textile types, monoterpenes, monoterpenes × concentrations, monoterpenes × textile types, concentrations × textile types and monoterpenes × concentrations × textile types on the IZ and the growth on textile samples after 14 days incubation with *A. flavus*, *F. culmorum*, and *A. niger*. Table 6 and Fig. 8 demonstrate the antifungal efficacy of eucalyptol and spathulenol against the development of *A. flavus*. When applied to wool fabric, eucalyptol at 50 µL/L revealed the maximum inhibition zone (6.33 mm), followed by eucalyptol at 50 µL/L (6 mm) when applied to cotton fabric. Eucalyptol at 50 µL/L applied to linen observed IZ (5.33 mm), spathulenol at 50 µL/L applied to wool revealed IZ of 5.333 mm, and spathulenol at 50 µL/L applied to cotton exhibited IZ value of 5 mm. After 14 days of incubation, eucalyptol and spathulenol at 50 µL/L and 25 µL/L revealed no development in the textile samples.

**Table 6:** Antifungal activity of eucalyptol and spathulenol-treated textiles against the growth of *A. flavus*, *F. culmorum*, and *A. niger* after 14 days incubation.

Compound	Conc. (µL/L)	Textile samples	*A. flavus*		*F. culmorum*		*A. niger*	
			IZ (mm)	Gr (mm)	IZ (mm)	Gr (mm)	IZ (mm)	Gr (mm)
Control	0	Linen	0	20	0	20	0	17.33 ± 2.51
		Cotton	0	17 ± 1	0	20	0	20
		Wool	0	20	0	20	0	20
		Silk	0	20	0	20	0	20
Eucalyptol spathulenol	50	Linen	5.33 ± 0.57	0	0	7.33 ± 1.52	5.33 ± 0.57	0
		Cotton	6 ± 1	0	0.33 ± 0.57	3 ± 2.64	5.33 ± 0.57	0
		Wool	6.33 ± 0.57	0	0.33 ± 0.57	2.33 ± 2.08	5.33 ± 0.57	0
		Silk	4.66 ± 0.57	0	0.33 ± 0.57	1.66 ± 1.52	5.33 ± 0.57	0
	25	Linen	4.33 ± 0.57	0	0	9.33 ± 0.57	4.33 ± 0.57	0
		Cotton	4.66 ± 0.57	0	0	1.66 ± 0.57	4.33 ± 0.57	0
		Wool	4.66 ± 0.57	0	0	5.33 ± 0.57	4.33 ± 0.57	0
		Silk	4 ± 1	0	0	5.33 ± 0.57	4.66 ± 0.57	0
	12	Linen	0.33 ± 0.57	0.33 ± 0.57	0	16.33 ± 0.57	1.33 ± 0.57	0
		Cotton	0.33 ± 0.57	0.66 ± 0.57	0	16 ± 1	1.33 ± 0.57	0
		Wool	0.33 ± 0.57	0.66 ±	0	16.66 ± 1.52	1.33 ± 0.57	0
		Silk	0.33 ± 0.57	0.66 ± 0.57	0	16.33 ± 0.57	1.33 ± 0.57	0
	6	Linen	0	1.66 ± 0.57	0	20	0.66 ± 1.15	0.66 ± 0.57
		Cotton	0	1.33 ± 0.57	0	19.66 ± 0.57	0.66 ± 0.57	0.33 ± 0.57
		Wool	0	2.66 ± 0.57	0	20	0.66 ± 0.57	0.33 ± 0.57
		Silk	0	1.33 ± 0.57	0	20	0.66 ± 1.15	0.66 ± 0.57
	50	Linen	4.66 ± 0.57	0	0	9.33 ± 0.57	4.66 ± 0.57	0
		Cotton	5 ± 1	0	0	1.33 ± 0.57	5	0
		Wool	5.33 ± 0.57	0	0	4 ± 1	5	0
		Silk	4.66 ± 0.57	0	0	10.33 ± 0.57	5.33 ± 0.57	0
	25	Linen	4.66 ±	0	0	10.33 ± 0.57	4.33 ± 0.57	0
		Cotton	4.33 ±	0	0	11.66 ± 0.57	4.66 ± 0.57	0
		Wool	4.66 ±	0	0	10.66 ± 0.57	4.33 ± 0.57	0
		Silk	4.33 ±	0	0	11.33 ± 1.15	4.33 ± 0.57	0
	12	Linen	0	1.33 ± 0.57	0	16.66 ± 1.15	1.33 ± 0.57	0
		Cotton	0	1.33 ± 0.57	0	16 ± 1	1.66 ± 0.57	0
		Wool	0	1.33 ± 0.57	0	15.33 ± 0.57	1.33 ± 0.57	0
		Silk	0	1	0	16 ± 1	1.33 ± 0.57	0
	6	Linen	0	3.66 ± 0.57	0	20	1 ± 1	0.33 ± ± 0.57
		Cotton	0	2.33 ± 0.57	0	20	1 ± 1	0.33 ± ± 0.57
		Wool	0	3.33 ± 0.57	0	20	0.33 ± 0.57	0.66 ± 0.57
		Silk	0	1.33 ± 0.57	0	20	1 ± 1	0.33 ± 0.57
*p*-value			0.9698	0.3508	0.9807	< 0.0001	0.9957	1.0000

Values are mean ± SD; IZ: Inhibition zone (mm); Gr: Growth on sample (mm).

**Figure 9:**
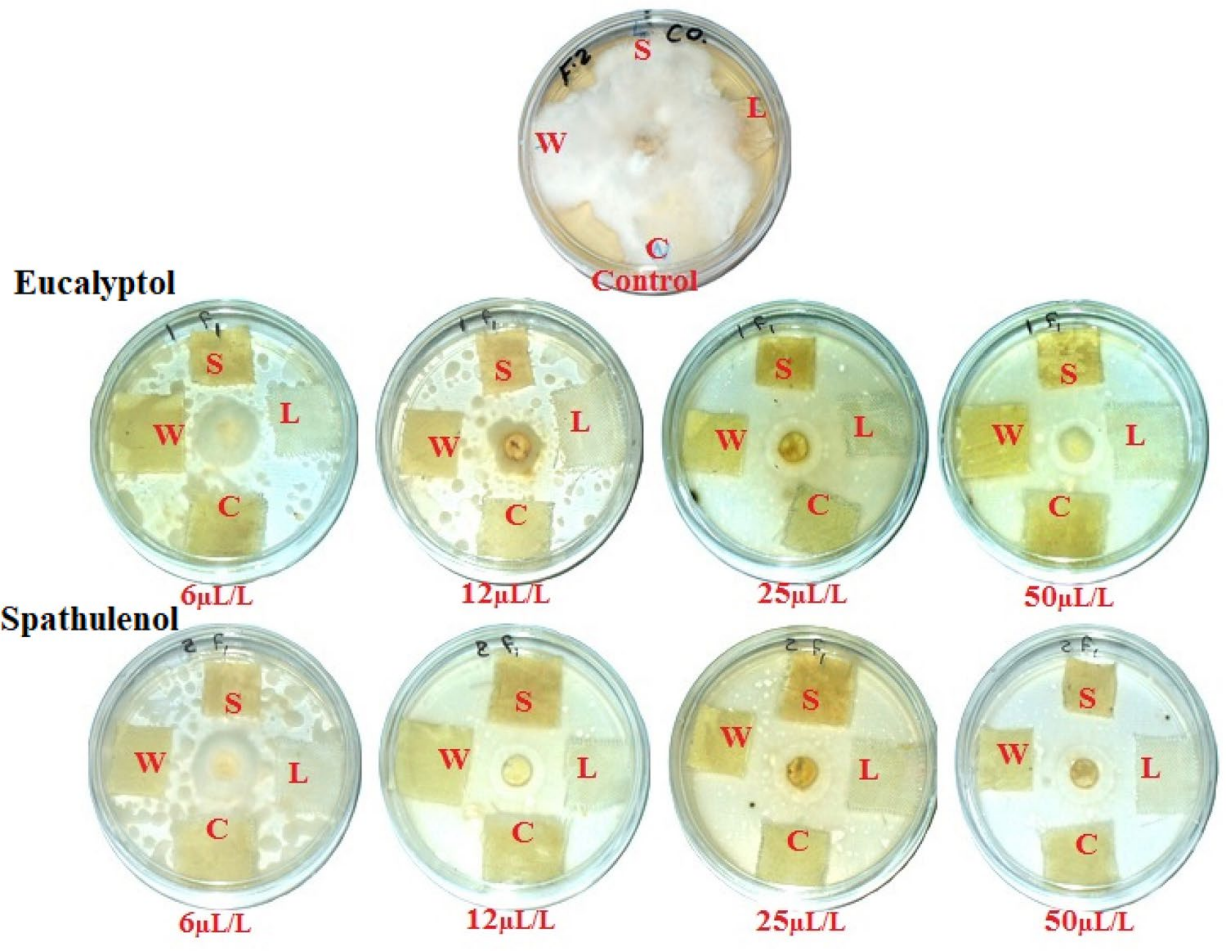
Visual observation of the effects of eucalyptol and spathulenol when treated textiles against the growth of *F. culmorum*. (Photos were taken by coauthor Wael A. A. Abo-Elgat). S: Silk; W: Wool; L: Linen; and C: Cotton.

Table 6 and Fig. 9 depict the antifungal action of eucalyptol and spathulenol against the development of *F. culmorum*. After 14 days, the four fabrics that had been exposed to both chemicals exhibited no signs of *F. culmorum* growth. The four fabrics treated with eucalyptol and spathulenol at 50, 25, and 12 µL/L exhibited no signs of microbial development. After 14 days of incubation, eucalyptol (applied at 50 µL/L to linen, cotton, wool, and silk) and spathulenol (applied at 50 µL/L to silk fabric) both demonstrated the highest IZ value (5.33 mm) against *F. culmorum* with no growth on them. Additionally, spathulenol revealed an IZ value of 5 mm against *F. culmorum* growth when applied to cotton and wool fibers at a rate of 50 µL/L.

**Figure 10:**
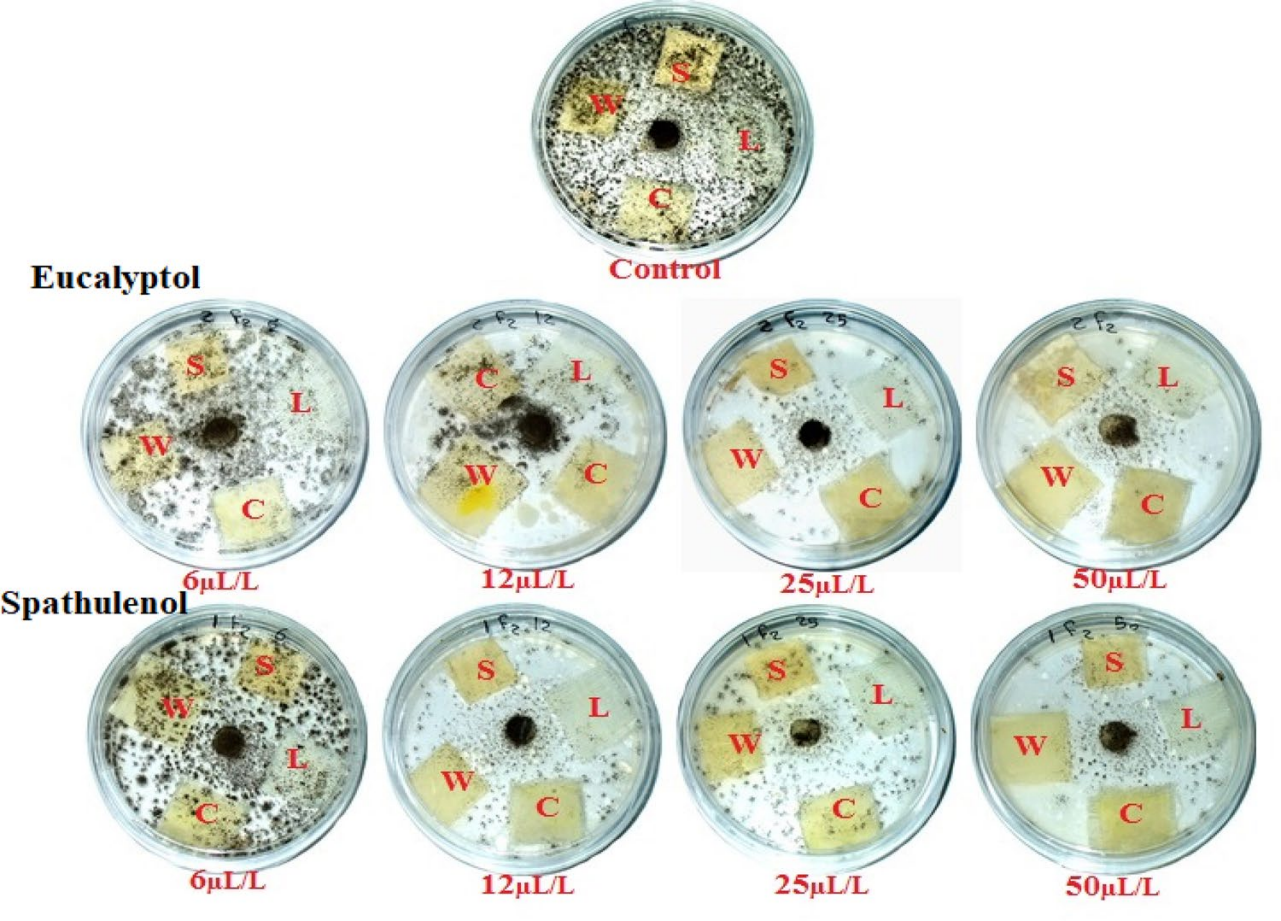
Visual observation of the effects of eucalyptol and spathulenol when treated textiles against the growth of *A. niger.* (Photos were taken by coauthor Wael A. A. Abo-Elgat). S: Silk; W: Wool; L: Linen; and C: Cotton.

Table 6 and Fig. 10 show the antifungal action of eucalyptol and spathulenol against the growth of *A. niger* when applied to four fabrics. Eucalyptol applied to cotton, wool, and silk at 50 µL/L demonstrated weak efficacy against *A. niger* growth, with minor IZ values (0.33 mm) mm detected. When applied to the four textiles where *A. niger* growth was at its strongest, other concentrations of eucalyptol and spathulenol showed little effect.

The minimum inhibitory concentrations (MICs) of the EO were observed to be 250, 500, and 250 µL/L, and 12, 50, and 2 µL/L when using the eucalyptol compound, while it was 25, > 50, and 2 µL/L when using spathulenol, against the growth of growth of *A. flavus*, *F. culmorum*, and *A. niger*, respectively.

The EO from *E. camaldulensis* and its bioactivity against the growth of three molds when applied to and functionalized on four textiles (linen, cotton, wool, and silk), as well as the main two compounds, eucalyptol and spathulenol, have been established and investigated based on the results presented above. When the EO evaluated by the GC–MS, the presence of primary bioactive chemicals were spathulenol, eucalyptol, *p*-cymene, crypton, carvacrol, phellandral, alloaromadendrene oxide-(2), 4-terpineol, cuminaldehyde, ylangenal, aromandendrene, *p*-cymen-7-ol, 4(15),5,10(14)-germacratrien-1-ol, and ledol.

All of the fabric samples indicated an increased quantity of fungi after just seven days. Other authors have already provided evidence of the potential intense development of fungi and bacteria on natural fiber fabrics, leading to their biodegradation^87–90^. The hyphae of fungi were seen under the SEM inspection in the untreated fabrics with EO and had a large amount of growth.. In the study by Crawford et al.^91^, SEM was used to observe the fracture planes and failure modes of the biocomposite samples as well as to see the hyphal growth of *Chaetomium globosum* on the natural fibers (non-woven flax, hemp, and oriented flax or hemp fiber (50%) and polypropylene fiber [50%]). Natural fibers can become deteriorated by microorganisms as a result of the production of hydrolytic extracellular enzymes such cellulase and lignase^92–94^. After a 35-day period of microbial incubation, flax fiber was definitely destroyed more than cotton fiber by the fungi *Trichoderma viride*, *Aspergillu niger*, and *Penicillium funiculosum*^95^. The majority of the fungi were recovered from the flax of the linen objects, which was further supported by their significant enzyme activity, particularly beta-glucosidase^96^. Selected isolates from historical textiles, including *Penicillium*, *Aspergillus*, and *Cladosporium*, also exhibited amylase activity, which is relevant since starch can be used to fill or glue textile fabrics^96^.

The growth of the molds *A. niger*, *T. viride*, and *Penicillium funiculosum* was found to be well-suited to cellulose fibers, and the bacterial growth of *Bacillus megaterium*, *Pseudomonas fluorescens*, and *Streptomyces* sp. to silk fabrics^95^. Compared to proteinaceous fibers, cellulose fibers are more sensitive to microbial breakdown. As a result, it's essential to maintain the necessary environmental conditions in museums because they cannot stop the growth of xerophilic fungi^96^. Since common airborne fungus species may pose a threat to textiles that have been preserved, using EOs and their primary constituents in vapor form may be an effective tactic to inhibit or prevent fungal growth. One great source for this is the EO from *E. camaldulensis* and its primary constituents.

The main compounds from *E. camaldulensis* EO were 1,8-cineole (eucalyptol), α-pinene, α-phellandrene, and p-cymene, which produced a complete inhibition to the mycelial growth of five *Fusarium* spp. at a concentration of 7–8 μL/mL after five days of incubation^97^. In another study, the use of *E. camaldulensis* EO prevented the growth of fungi that colonized as wood rot fungi, household molds, or plant pathogenic fungi including* C. globosum*, *Fusarium oxysporum*,* A. niger*, *Thanatephorus cucumeris,* and *Rhizopus oryzae*^98^. At a concentration of 5 mg/mL of *E. camaldulensis* EO, the mycelial growth of *F. oxysporum* was suppressed to 84% of its maximum potential^98^. With neither p-cymene or cryptone discovered in the plant collected from Taiwan, the leaf EO of *E. camaldulensis* revealed the existence of 1,8-cineole as a dominating compound and demonstrated fungal growth inhibition against *A. clavatus* and *A. niger* at 79.66% and 24.05%, respectively, after 7 days of inoculation^99^. At 20 μL/plate, the EOs from eleven *E. camaldulensis* from Sardinia, Italy, totally inhibited *A. flavus*. These compounds included *p*-cymene, 1,8-cineole, *β*-phellandrene, spathulenol and cryptone^100^. The primary compounds found in *E. camaldulensis* EO with significant antifungal activity against *F. culmorum* and *F. solani* were p-cymene, γ-eudesmol, L-linalool, and piperitone^101^.

The 1,8-cineole was found as the primary compound in the EOs of *E. largiflorens*, *E. microtheca*, *E. oleosa*, *E. spathulata* and *E. torquata*, and they showed substantial antifungal activity against *Aspergillus flavus*, *A. parasiticus*, *A. niger*, *Penicillium chryzogenum*, and *P. citrinum*^102^. When applied to some wood samples, *E. camaldulensis* leaf EO, which primarily contains eucalyptol, α-pinene, and γ-terpinene demonstrated powerful antifungal activity against *C. globosum* no inhibition against *A.* *niger*, and *Trichoderma* *viride* and only minimal inhibition of *Fusarium* *subglutinans* at high concentration^72^.

The amount of 1,8-cineole, *p*-cymene, *α*-pinene, and cryptone and the antibacterial activity were found to be somewhat correlated^103^. The increase *p*-cymene concentration in leaf EO may be the cause of its antimicrobial activity^104^. According to studies by Bowers and Locke^105^, Inouye et al.^106^, and Angelini et al.^107^, *Melaleuca alternifolia* EO has an inhibitory effect on phytopathogenic fungi like *A. fumigatus*, *F. solani*, *P. expansum*, *Botrytis cinerea*, *R. oryzae*, *Blumeria graminis*, and *Pyrenophora graminea*. Two mycotoxigenic fungi, *F. graminearuma*, and *F. culmorum*, were subjected to the effects of EO and its main purified components (terpinen-4-ol, terpinen, and 1,8-cineole)^108^.

The biocidal (antibacterial, antiviral, antifungal and insect repellent) uses and therapeutic benefits are possible with the EO-based bio-functional textiles^109,110^. Using 1%, 3%, and 5% of each EO from rosemary and orange which contain the abundant compounds (eucalyptol, camphor, and *α*–pinene), and (limonene, limonene oxide, *α*–pinene, and *β*-phellandrene), respectively, treated textile substrate (56% cotton/44% polyester) reduced rates of 22.12%, and 51.45% against *A. niger*, and 18.3%, and 60.57% against *A. flavus*, respectively^61^.

Application of EO from Thyme applied to linen-cotton blended fabric at an 8% concentration in methanol revealed possible antimicrobial activity with no mold growth and or appreciable loss of braking force^111^. The growth of *A. niger* mold could be completely inhibited by applying 2% to 25% of lemongrass EO on polyester fabric discs^112^. The best antimicrobial activity against *Staphylococcus aureus*, *Klebsiella Pneumoniae* and *Candida albicans* is also seen in treated or colored cotton, wool, silk, and nylon fabrics that have curcumin extract added^25^.

With an acrylate-based binder to secure the microcapsules to the fabric, rose and sage EO microcapsules were applied to 100% cotton and 50% cotton/50% polyester woven fabrics, respectively, and demonstrated improved fabric durability through washing and handling as well as good candidates for providing biological properties^113^. When applied to fabrics, the EOs from Patchouli^114,115^, *Artemsia argyi*^116^ and moxa^117^ exhibits eco-friendly antibacterial action. Cotton fabric treated with rosemary has shown effective antibacterial action against *Staphylococcus aureus* and *Escherichia coli*^118^. *Cinnamomum zeylanicum* EO-functionalized heritage textiles using vapor phase showed good antimicrobial activities against *A. niger*, *Penicillium funiculosum*, *Trichoderma viride*, *Streptomyces rutgersensis*, *Bacillus megaterium*, and *Pseudomonas fluorescens*^119^.

The activity of the EO could be related to the main compounds eucalyptol and spathulenol. Eucalyptol was observed toxic effects on in vitro mycelium growth of *F. subglutinans*, *F. cerealis*, *F. verticillioides*, *F. proliferatum*, *F. oxysporum*, *F. sporotrichioides*, *A. tubingensis*, *A. carbonarius*, *Alternaria alternata* and *Penicillium* sp.^120^. Eucalyptol reduced the growth of plant pathogenic fungus *Botrytis fabae* significantly^121^. Eucalyptol was shown to have comparable fungicide properties against *F. oxysporum* f. sp. *albedinis*^122^. Spathulenol was observed potential activity against several yeasts and filamentous fungi like *Tricophyton mentagrophytes* and *Microsporum gypseum*^123^. The EO of *Psidium guineense* and spathulenol were observed potential antioxidant, anti-inflammatory, antiproliferative and antimycobacterial activities^124^.

It is significant to remember that in vivo toxicity is not necessarily equivalent to in vitro bioactivity data. This work thus paves the way for future investigations into the effects of medicines over a lengthy period of time, or what is known as shelf life. study of the speeds at which oil vapors employed in treatments evaporate and are absorbed by textile fibers. Future research is advised to measure the characteristics of textile fibers and the degree to which treatments alter them, as well as the application of treatments to numerous other types of textile fibers. Studying mixes of oils in various proportions and concentrations, as well as researching various application techniques, can help us better understand various types and sources of oils as well as their biological resistance capabilities.

One of the future visions of the objectives of the study is the extent to which natural extracted oils can be applied in protecting ancient textiles preserved in museums, libraries, and inside display cases. Using fumigation of the extracted materials inside the displays to eliminate biological growths. While providing future protection for the textiles from the effects of fungal damage.

## Conclusions

The EO from *E. camaldulensis* leaves and its two primary components, eucalyptol and spathulenol, were used to bio-functionalize four fabrics (linen, cotton, wool, and silk). Spathulenol molecule has the highest electrostatic potential, followed by eucalyptol, according to quantum chemistry calculations. The EO and the two major chemicals were specifically tested for their capacity to inhibit the growth of the three typical fungal contaminants of textiles, *Aspergillus flavus*, *Fusarium culmorum*, and *Aspergillus niger*. The vapor phase of *E. camaldulensis* EO and eucalyptol demonstrated a potent inhibitor of fungal growth. This indicates the potential for using EO or eucalyptol as natural biocides during in vitro and in situ applications to prevent the growth of fungi by substituting the most prevalent toxic biocide typically employed in the conservation of cultural material stored in libraries and museums, such as textiles. Subsequently and since all of the experiments were conducted in vitro, other variables like temperature and relative humidity on a wide scale would undoubtedly have an effect on the development and other characteristics of the textile. As a result, this study will allow for further investigation to determine the effect of essential oils as effective antimicrobials on the conserved antique textiles in museums throughout the world.

### Supplementary Information

Supplementary Information.

## Data Availability

All data generated or analyzed during this study are included in this published article.
