# Supplementary material for: GC–MS, quantum mechanics calculation and the antifungal activity of river red gum essential oil when applied to four natural textiles
Source: Sci Rep. 2023 Oct 25;13:18214. doi: 10.1038/s41598-023-45480-x (PMC10600096; doi:10.1038/s41598-023-45480-x)
Supplement: Supplementary file 1 — Supplementary Information. [file 41598_2023_45480_MOESM1_ESM.docx]

**GC-MS, quantum mechanics calculation and the antifungal activity of river red gum essential oil when applied to four natural textiles**

Ayman S. Taha ^a^, Ibrahim H. M. Ibrahim ^b^, Wael A. A. Abo-Elgat ^b^, Ahmed Abdel-Megeed ^c^, Mohamed Z. M. Salem ^d,^*, Mamoun S. M. Abd El-Kareem ^e^

^a^ *Conservation Department, Faculty of Archaeology, Aswan University, Aswan 81528, Egypt;*

[*AymanSalah@arc.aswu.edu.eg*](mailto:AymanSalah@arc.aswu.edu.eg)

^b^ *Restoration Department, High Institute of Tourism, Hotel Management and Restoration, Abu Qir, Alexandria, Egypt;* [*ibrahim_elkholy88@yahoo.com*](mailto:ibrahim_elkholy88@yahoo.com)*;* [*watsat20@yahoo.com*](mailto:watsat20@yahoo.com)

*^c^ Department of Plant Protection, Faculty of Agriculture (Saba Basha), Alexandria University,*

*Alexandria 21531, Egypt; hekemdar@yahoo.com*

^d^ *Forestry and Wood Technology Department, Faculty of Agriculture (EL-Shatby), Alexandria University, Alexandria, Egypt;* [mohamed-salem@alexu.edu.eg](mailto:mohamed-salem@alexu.edu.eg)

*^e^ Atomic and Molecular Physics Unit, Experimental Nuclear Physics Department, Nuclear Research Centre, Egyptian Atomic Energy Authority, Inshas, Cairo 13759, Egypt;* [*mamoun_sarhan@yahoo.com*](mailto:mamoun_sarhan@yahoo.com)

* Correspondence: [mohamed-salem@alexu.edu.eg](mailto:mohamed-salem@alexu.edu.eg)

**
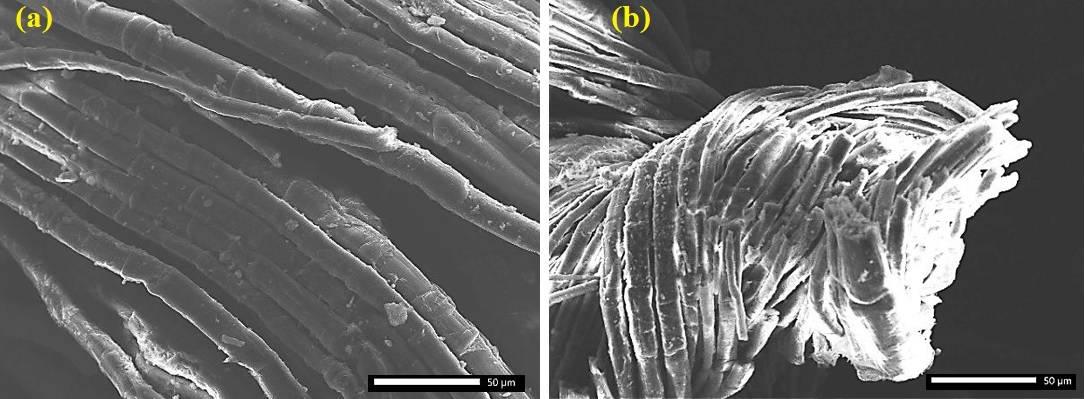
**

**
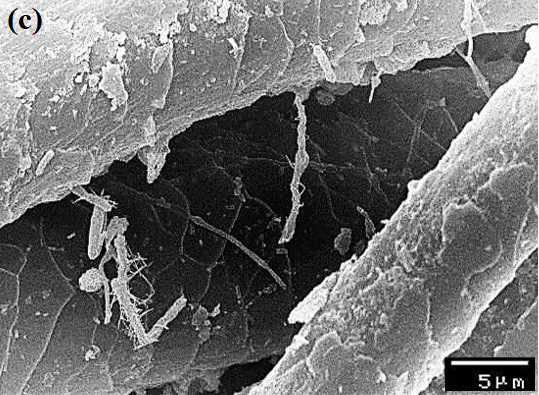
**

**
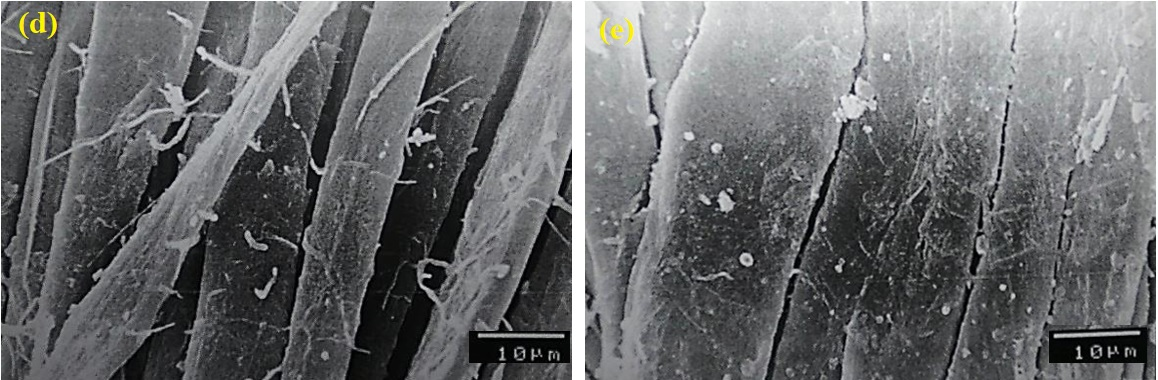
**

**
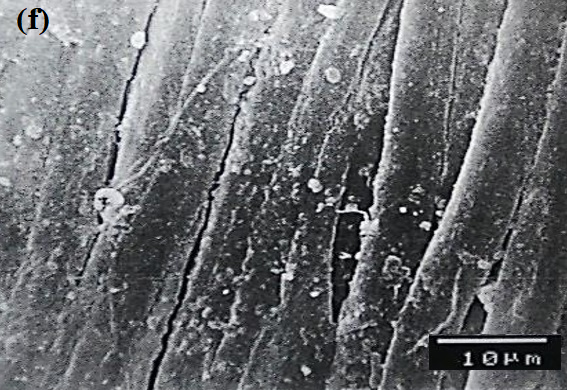
**

**Figure S1.** Displays the SEM micrographs of ancient textiles in longitudinal view. Linen warp from the Coptic fabric (a), Undyed linen weft from the Coptic fabric (b), Dyed wool weft from the Coptic fabric (c), Cotton warp from the Islamic woven (d), Cotton weft from the Islamic woven (e), and Woven inscription silk yarn from the Islamic woven (f).

**
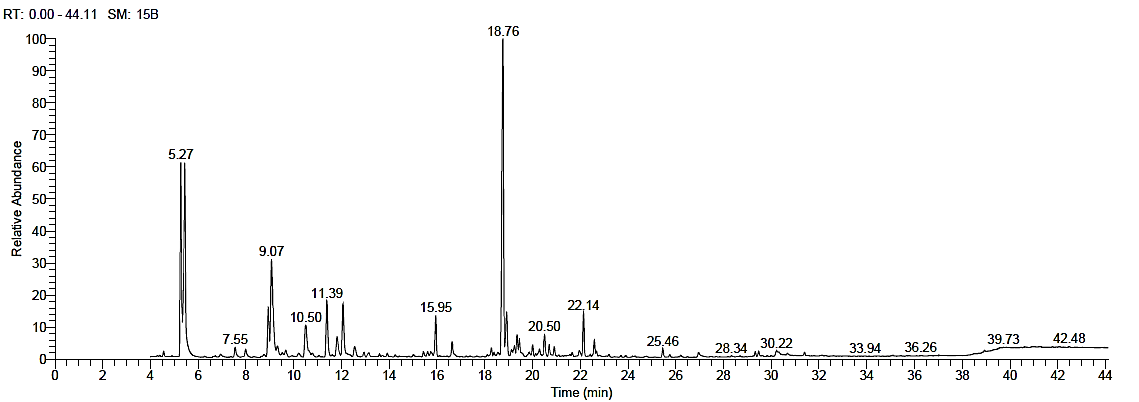
**

**Figure S2.** GC-MS chromatogram of the chemical components found in *E. camaldulensis* essential oil


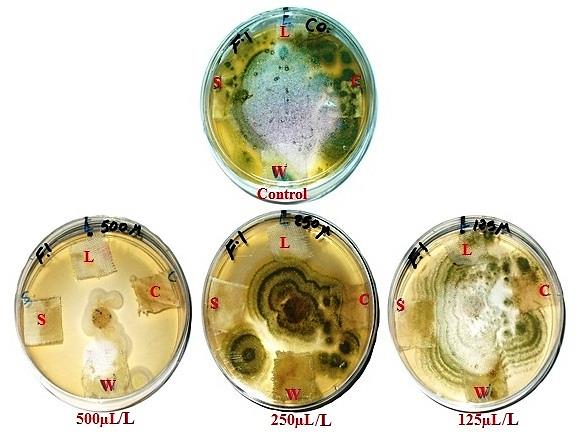


**Figure S3.** Visual observation of antifungal activity of *Eucalyptus camaldulensis* EO applied to the textiles (L: linen– C: cotton- W: wool– S: silk) by vapor method at 500, 250, and 125 µL/L against *A. flavus* (Photos were taken by coauthor Wael A. A. Abo-Elgat).

**
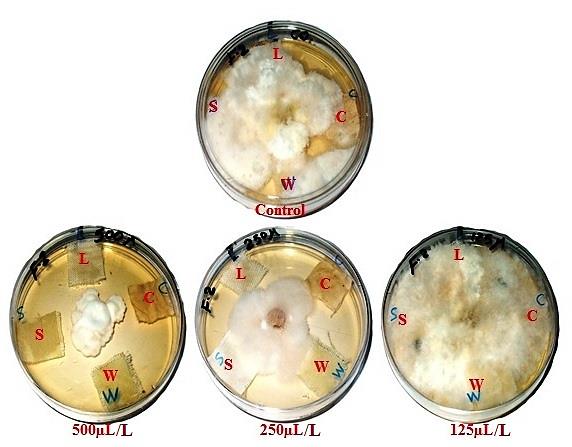
**

**Figure S4.** Visual observation of antifungal activity of *E. camaldulensis* EO applied to the textiles (L: linen– C: cotton- W: wool– S: silk) by vapor method at 500, 250, and 125 µL/L against *F. culmorum* growth (Photos were taken by coauthor Wael A. A. Abo-Elgat).


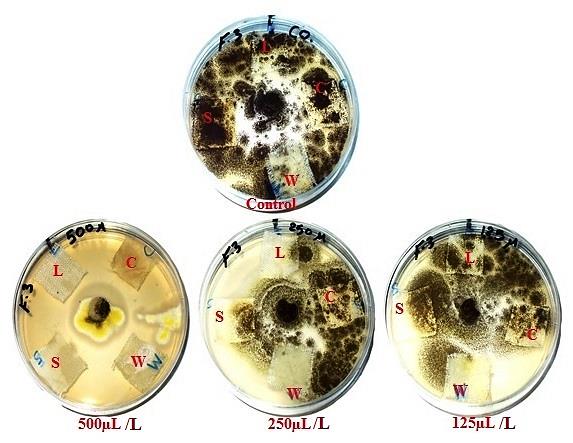


**Figure S5.** Visual observation of the antifungal activity of *Eucalyptus camaldulensis* EO, applied to the textiles (L: linen– C: cotton- W: wool– S: silk) by vapor method at 500, 250, and 125 µL/L against *A. niger* (Photos were taken by coauthor Wael A. A. Abo-Elgat).

**Table S1.** The biological effects of *E. camaldulensis* EO concentrations

| Inhibition zone (mm) 7th day against *Aspergillus flavus* | | | | | |
| --- | --- | --- | --- | --- | --- |
| Source | DF | Type III SS | Mean Square | F Value | Pr > F |
| Concentrations (A) | 3 | 181.5 | 60.5 | 103.71 | <0.0001 |
| Treatments (B) | 3 | 30 | 10 | 17.14 | <0.0001 |
| A * B | 9 | 57.5 | 6.38 | 10.95 | <0.0001 |
| Inhibition zone (mm) 14th day against *Aspergillus flavus* | | | | | |
| A | 3 | 128.666 | 42.88 | 257.33 | <0.0001 |
| B | 3 | 15.5 | 5.166 | 31.00 | <0.0001 |
| A * B | 9 | 46.5 | 5.166 | 31.00 | <0.0001 |
| Growth of *Aspergillus flavus* on sample (mm) 7th day | | | | | |
| A | 3 | 801.062 | 267.02 | 261.57 | <0.0001 |
| B | 3 | 17.72 | 5.909 | 5.79 | 0.0028 |
| A * B | 9 | 31.52 | 3.502 | 3.43 | 0.0047 |
| Growth of *Aspergillus flavus* on sample (mm) 14th day | | | | | |
| A | 3 | 2718.395 | 906.13 | 543.68 | <0.0001 |
| B | 3 | 15.562 | 5.18 | 3.11 | 0.0399 |
| A * B | 9 | 14.02 | 1.55 | 0.93 | 0.5093 |
| Inhibition zone (mm) 7th day against *Fusarium culmorum* | | | | | |
| A | 3 | 158.25 | 52.75 | 180.86 | <0.0001 |
| B | 3 | 2.083 | 0.69 | 2.38 | 0.0879 |
| A * B | 9 | 12.25 | 1.36 | 4.67 | 0.0005 |
| Inhibition zone (mm) 14th day against *Fusarium culmorum* | | | | | |
| A | 3 | 126.562 | 42.18 | 253.12 | <0.0001 |
| B | 3 | 2.229 | 0.743 | 4.46 | 0.0100 |
| A * B | 9 | 6.687 | 0.743 | 4.46 | 0.0008 |
| Growth of *Fusarium culmorum* on sample (mm) 7th day | | | | | |
| A | 3 | 3374.729 | 1124.909 | 760.50 | <0.0001 |
| B | 3 | 52.56 | 17.52 | 11.85 | <0.0001 |
| A * B | 9 | 147.354 | 16.372 | 11.07 | <0.0001 |
| Growth of *Fusarium culmorum* on sample (mm) 14th day | | | | | |
| A | 3 | 4329.56 | 1443.187 | 4618.20 | <0.0001 |
| B | 3 | 4.729 | 1.576 | 5.04 | 0.0056 |
| A * B | 9 | 7.187 | 0.798 | 2.56 | 0.0244 |
| Inhibition zone (mm) 7th day against *Aspergillus niger* | | | | | |
| A | 3 | 372.56 | 124.187 | 745.12 | <0.0001 |
| B | 3 | 5.229 | 1.743 | 10.46 | <0.0001 |
| A * B | 9 | 19.354 | 2.150 | 12.90 | <0.0001 |
| Inhibition zone (mm) 14th day against *Aspergillus niger* | | | | | |
| A | 3 | 232.56 | 77.52 | 531.57 | <0.0001 |
| B | 3 | 3.56 | 1.187 | 8.14 | 0.0004 |
| A * B | 9 | 10.68 | 1.187 | 8.14 | <0.0001 |
| Growth of *Aspergillus niger* on sample (mm) 7th day | | | | | |
| A | 3 | 1492.72 | 497.576 | 174.33 | <.0001 |
| B | 3 | 161.729 | 53.909 | 18.89 | <.0001 |
| A * B | 9 | 389.68 | 43.298 | 15.17 | <.0001 |
| Growth of *Aspergillus niger* on sample (mm) 14th day | | | | | |
| A | 3 | 2691.89 | 897.298 | 897.30 | <0.0001 |
| B | 3 | 256.229 | 85.409 | 85.41 | <0.0001 |
| A * B | 9 | 430.354 | 47.817 | 47.82 | <0.0001 |
| Note: Concentrations are 500, 250, 125 and 0 µL/L; treatments are linen, cotton, wool, and silk-treated EO | | | | | |

**Table S2.** The biological effects of the monoterpenes and their concentrations

| Inhibition zone (mm) 14th day against *Aspergillus flavus* | | | | | |
| --- | --- | --- | --- | --- | --- |
| Source | DF | Type III SS | Mean Square | F Value | Pr > F |
| Concentrations (A) | 4 | 671.13 | 167.783 | 774.38 | <.0001 |
| Monoterpenes (B) | 1 | 1.008 | 1.008 | 4.65 | 0.0340 |
| Textile type (C) | 3 | 1.82 | 0.608 | 2.81 | 0.0448 |
| A*B | 4 | 2.36 | 0.59 | 2.73 | 0.0347 |
| A*C | 12 | 3.80 | 0.31 | 1.46 | 0.1565 |
| B*C | 3 | 0.49 | 0.16 | 0.76 | 0.5219 |
| A*B*C | 12 | 0.96 | 0.08 | 0.37 | 0.9698 |
| Growth of *Aspergillus flavus* on sample (mm) 14th day | | | | | |
| Concentrations (A) | 4 | 6627.63 | 1656.908 | 9468.05 | <.0001 |
| Monoterpenes (B) | 1 | 3.008 | 3.008 | 17.19 | <.0001 |
| Textile type (C) | 3 | 12.49 | 4.16 | 23.79 | <.0001 |
| A*B | 4 | 4.70 | 1.17 | 6.71 | 0.0001 |
| A*C | 12 | 38.63 | 3.219 | 18.40 | <.0001 |
| B*C | 3 | 1.091 | 0.36 | 2.08 | 0.1095 |
| A*B*C | 12 | 2.36 | 0.19 | 1.13 | 0.3508 |
| Inhibition zone (mm) 14th day against *Fusarium culmorum* | | | | | |
| Concentrations (A) | 4 | 0.3 | 0.075 | 3.00 | 0.0232 |
| Monoterpenes (B) | 1 | 0.07 | 0.075 | 3.00 | 0.0871 |
| Textile type (C) | 3 | 0.025 | 0.008 | 0.33 | 0.8013 |
| A*B | 4 | 0.3 | 0.075 | 3.00 | 0.0232 |
| A*C | 12 | 0.1 | 0.008 | 0.33 | 0.9807 |
| B*C | 3 | 0.02 | 0.008 | 0.33 | 0.8013 |
| A*B*C | 12 | 0.1 | 0.008 | 0.33 | 0.9807 |
| Growth of *Fusarium culmorum* on sample (mm) 14th day | | | | | |
| Concentrations (A) | 4 | 4611.21 | 1152.804 | 1554.34 | <.0001 |
| Monoterpenes (B) | 1 | 76.8 | 76.8 | 103.55 | <.0001 |
| Textile type (C) | 3 | 67.5 | 22.5 | 30.34 | <.0001 |
| A*B | 4 | 153.616 | 38.404 | 51.78 | <.0001 |
| A*C | 12 | 104.916 | 8.743 | 11.79 | <.0001 |
| B*C | 3 | 20.33 | 6.77 | 9.14 | <.0001 |
| A*B*C | 12 | 127.58 | 10.63 | 14.34 | <.0001 |
| Inhibition zone (mm) 14th day against *Aspergillus niger* | | | | | |
| Concentrations (A) | 4 | 509.7 | 127.42 | 364.07 | <.0001 |
| Monoterpenes (B) | 1 | 0.008 | 0.008 | 0.02 | 0.8778 |
| Textile type (C) | 3 | 0.425 | 0.141 | 0.40 | 0.7500 |
| A*B | 4 | 0.86 | 0.216 | 0.62 | 0.6502 |
| A*C | 12 | 0.7 | 0.058 | 0.17 | 0.9992 |
| B*C | 3 | 0.291 | 0.097 | 0.28 | 0.8413 |
| A*B*C | 12 | 1 | 0.083 | 0.24 | 0.9957 |
| Growth of *Aspergillus niger* on sample (mm) 14th day | | | | | |
| Concentrations (A) | 4 | 7095.5 | 1773.87 | 4627.50 | <.0001 |
| Monoterpenes (B) | 1 | 0.008 | 0.008 | 0.02 | 0.8832 |
| Textile type (C) | 3 | 6.158 | 2.052 | 5.36 | 0.0021 |
| A*B | 4 | 0.033 | 0.008 | 0.02 | 0.9991 |
| A*C | 12 | 25.96 | 2.163 | 5.64 | <.0001 |
| B*C | 3 | 0.091 | 0.03 | 0.08 | 0.9708 |
| A*B*C | 12 | 0.366 | 0.03 | 0.08 | 1.0000 |
| Note: Concentrations are 0 μL/L, 6 μL/L, 12 μL/L, 25 μL/L, and 50 μL/L; textile types are linen, cotton, wool, and silk-treated EO and monoterpenes are eucalyptol and spathulenol. | | | | | |
